# Household demand persistence for child micronutrient supplementation

**DOI:** 10.1016/j.jhealeco.2018.09.010

**Published:** 2018-11

**Authors:** Travis J. Lybbert, Stephen A. Vosti, Katherine P. Adams, Rosemonde Guissou

**Affiliations:** a University of California, Davis, United States; b IRSS, Burkina Faso

**Keywords:** Undernutrition, Early childhood, Micronutrients, Supplementation, Demand, Burkina Faso

## Abstract

Addressing early-life micronutrient deficiencies can improve short- and long-term outcomes. In most contexts, private supply chains will be key to effective and efficient preventative supplementation. With established vendors, we conducted a 60-week market trial for a food-based micronutrient supplement in rural Burkina Faso with randomized price and non-price treatments. Repeat purchases – critical for effective supplementation – are extremely price sensitive. Loyalty cards boost demand more than price discounts, particularly in non-poor households where the father is the cardholder. A small minority of households achieved sufficient supplementation for their children through purely retail distribution, suggesting the need for more creative public-private delivery platforms informed by insights into household demand persistence and heterogeneity.

## 1 Introduction

Recent advances in our understanding of the irreversible, deleterious effects of early childhood undernutrition on cognitive and physical development, human capital accumulation, and adult economic productivity have inspired a coordinated global commitment to preventing undernutrition during the critical first 1000 days of life (Hoddinott et al., 2013; Sharma et al., 2017; Shekar et al., 2017; Victora et al., 2008). Micronutrient supplementation has emerged as a particularly promising element of efforts aimed at preventing these undernutrition deficits (Bhutta et al., 2013). Like other preventative health products, however, micronutrient supplements raise several delivery challenges stemming largely from limited private demand among households most vulnerable to malnutrition. Many of the preventative health products featured in recent research in this literature exploring private demand are durable goods such as insecticide-treaded bed nets, latrine slabs and rubber shoes (Dupas, 2011; Dupas and Miguel, 2017; Meredith et al., 2013; Peletz et al., 2017; Tarozzi et al., 2014). In contrast, micronutrient supplementation requires high-frequency (e.g., daily) consumption, which raises different and more complex demand- and supply-side considerations and further amplifies delivery challenges. To shed light on this delivery dilemma – which hinges crucially on private supply chains and private demand – we use a market experiment to estimate the determinants and dynamics of household demand for a preventative food-based micronutrient supplement inspired by a revolutionary therapeutic nutrition product.

Since the early 2000s, a fortified peanut butter-based product called Plumpy’Nut® has seen remarkable success as a *treatment* for severe acute malnutrition (SAM) among young children. Showcased widely in media outlets, this success prompted several companies to formulate similar energy-dense products that also provide micronutrients and essential fatty acids in a lipid-based (typically, peanut) paste – products collectively known as Ready-To-Use Therapeutic Foods (RUTF) or ‘large-quantity’ Lipid-based Nutrient Supplements’ (LNS). Potent anecdotal and more rigorous evidence of the success and cost-effectiveness of LNS products in treating SAM accumulated rapidly (Ciliberto et al., 2005; Diop el et al., 2003).^1^

This unprecedented success, coupled with increasing recognition of undernutrition as a top global health and economic development priority (Hoddinott et al., 2013), has stimulated the emergence of a globally-recognized, coordinated framework for addressing undernutrition. At its center, the Scaling Up Nutrition (SUN) movement advocates multi-sectoral strategies to reduce undernutrition as complements to direct nutrition interventions such as therapeutic feeding, food fortification, micronutrient supplementation, and breastfeeding promotion (Nabarro et al., 2012; Scaling Up Nutrition, 2012). While the effectiveness of RUTF in treating SAM is clear, greater awareness of the irreversible, long-term deleterious effects of less life-threatening forms of undernutrition has spawned broad recognition of the need not only to treat children suffering from SAM but also to *prevent* undernutrition during the critical first 1000 days of life (Arimond et al., 2013). Sustainable, long-term strategies to prevent undernutrition, including largely invisible micronutrient deficiencies described as ‘hidden hunger’, will likely be primarily food-based (e.g., dietary diversification and food fortification) (von Grebmer et al., 2014). In the short-term, however, supplementation can help meet the high nutrient requirements of pregnant and lactating mothers and of children from 6 to 24 months of age (Arimond et al., 2013; Dewey and Vitta, 2012). Fueled by this need and the success of large- quantity LNS, nutritionists and food scientists have designed small-quantity LNS (SQ-LNS) products to prevent undernutrition. Clinical trial results in our study area in Burkina Faso show that these SQ-LNS products, when provided with monitoring and treatment of malaria and diarrhea to children from 6 to 18 months of age, improved linear growth, decreased the prevalence of both stunting and wasting, and positively affected some aspects of cognitive and behavioral development (Hess et al., 2017, 2015; Prado et al., 2016). Extrapolating the estimated impact on stunting alone suggests that scaling up access to SQ-LNS with similar usage rates nationwide could save more than 25,000 young lives in Burkina Faso over the next ten years.

Despite apparent similarities, differences in these LNS products raise important supply chain issues. Large-quantity LNS products are designed to be consumed intensively by children as a short-term emergency treatment regimen and are exclusively distributed via public channels. Indeed, in many countries, including Burkina Faso, it is illegal for individuals to buy or sell these products. In contrast, SQ-LNS products are designed to be consumed daily for extended periods of time to guard against undernutrition – particularly among young children and pregnant/lactating women. These differences have profound implications for supply chains. Whereas organizations such as UNICEF, World Food Programme, and *Médecins Sans Frontièrs* have purchased and delivered most of the large-quantity LNS and coordinated key supply chain relationships and regulations, they are unlikely to similarly take the lead on widespread distribution of SQ-LNS products (Lybbert, 2011). Instead, any coordination that emerges among stakeholders and supply chains is likely to involve a mix of public and private sector engagement and, in order to reach children most at-risk for hidden hunger, a blend of market and health clinic distribution.^2^

Where and how such a hybrid distribution system emerges will depend on a host of site-specific factors, including nutritional needs, private sector capacity to produce and distribute these products, public health and other infrastructure, public sector commitments to necessary investments and coordination, and – crucially – private demand for SQ-LNS products in a broader context of household food, nutrition and health choices (Dupas, 2011; Dupas and Miguel, 2017; Meredith et al., 2013). Demand for preventive products like SQ-LNS is, in theory, determined by the product’s discounted stream of private costs and benefits. However, liquidity constraints, insufficient or incomplete information, limited education, and present-biased preferences can stifle demand in practice. Rigorous experimental evidence on demand for preventative health products raises several such issues. Unlike curative or therapeutic health products, demand for most preventative health products is very price sensitive, is often low even at highly subsidized prices, and can be shaped by non-price factors such as commitment devices and reminders (Dupas and Miguel, 2017).

Among preventative products, two dimensions – procurement frequency and impact detectability – crucially determine the drivers and dynamics of private demand. Many influential studies in this literature focus on relatively durable health investments for which a single procurement decision enables frequent and sustained use and provides a stream of expected benefits that spans many weeks or months (e.g., Cohen and Dupas, 2010; Dupas, 2014, 2009, Kremer and Miguel, 2007; Meredith et al., 2013; Tarozzi et al., 2014). In contrast, preventative health products that are nondurable in the sense that use requires outright consumption – such as chlorine, soap and vitamins, all of which have featured in recent demand studies (e.g., Ashraf et al., 2010; Meredith et al., 2013) – entail a categorically different demand decision as households must synchronize much more closely their procurement and consumption frequency in order to ensure sustained access.^3^ Along with procurement frequency, the detectability of impacts of preventative health products also importantly shape private demand. The benefits associated with products that improve sanitation or prevent disease, for example, are more readily detectable by beneficiaries (or their caregivers) than those that improve longer run and often more subtle health outcomes associated with physical growth or cognitive development. Because consumers can more easily connect their use of products such as latrine slabs and bed nets to observable private benefits than they can with micronutrient supplementation, demand for the latter may be weaker and more sensitive to price and non-price factors.

In this broader preventative product context, SQ-LNS is characterized by high procurement frequency and low impact detectability, which likely weakens private demand and raises novel challenges for identifying viable delivery mechanisms. Rather than simply documenting limited household demand for SQ-LNS, our aim in this study is to elucidate the dynamics of demand in order to size up options for tapping more completely the considerable demonstrated long-term benefits of SQ-LNS. Understanding the determinants of demand persistence for SQ-LNS sets the stage for designing, testing and scaling up production and delivery options and addressing cost-sharing issues. Specifically, by shaping which households purchase these products, how regularly children consume them, and how these consumption patterns translate into real health benefits, private demand for SQ-LNS products will determine the viability and sustainability of alternative hybrid public-private options for delivering daily supplements to the urban and rural poor. Household demand for these products thereby creates or constrains opportunities to leverage the comparative advantage and resources of public organizations and private firms, respectively.

We created an experimental market for SQ-LNS in rural Burkina Faso to shed light on private demand and associated implications for the feasibility of market-based delivery. We take the recent and rigorous evidence of the encouraging child growth effects of SQ-LNS supplementation in this setting (Hess et al., 2017) and the potential impact of scaling such an intervention as the point of departure for our experimental markets design. Beginning in 2013, we conducted experimental auctions for SQ-LNS in our study area. These auctions, which included some households that participated in the clinical trial but primarily consisted of other households with children in the target age range (6–24 months), enable us to estimate complete, incentive-compatible demand curves for SQ-LNS. Immediately after conducting the auctions, we launched a market trial with 32 vendors in these villages. Since the persistence of dynamics of demand is our main objective, we intentionally set prices in the range of target households’ expected willingness-to-pay rather than higher levels required to make pure retail markets viable. An initial village-level randomization market trial allows us to estimate the price elasticity of demand for SQ-LNS overall, as well as for initial and repeat purchases separately. A subsequent parent randomization enables us to estimate the demand effects of a loyalty card that offers a small reward to households that purchase a complete month’s supply of SQ-LNS, including differentiated effects of this loyalty card based on whether the mother or father is the cardholder. Using high-resolution rainfall data and detailed endline data for a sub-sample of households, we are also able to estimate the sensitivity of demand to departures from normal rainfall and to selected household characteristics.

Using this experimental design, we provide the first rigorous evaluation of the dynamics and persistence of household demand for a nutritional supplement.^4^ We find that price elasticity of demand for SQ-LNS is high on average, but especially high for repeat purchases – the crux of any effective, sustainable supplementation strategy. Even if SQ-LNS is cost-effective as a nutritional investment from a societal perspective, private demand may cover less than half the production and distribution costs of SQ-LNS, suggesting the need for mixed private-public delivery strategies and cost-sharing arrangements. Second, we estimate habit formation and supplementation ‘survival’ models to unpack the effects of price and non-price factors on demand. We find evidence of habit formation, particularly among non-poor households. We further find evidence that a simple loyalty card induces a much larger demand response than a price discount of roughly the same value, suggesting important behavioral influences. Finally, based on an endline survey of a subset of households, we find that demand persistence is highest in wealthy households that purchase half or more of their food in markets. Understanding the determinants, dynamics, and persistence of household demand for supplementation in these ways can directly inform the design of delivery models for micronutrient supplements such as SQ-LNS. While these food-based supplements are a potentially potent remedy for hidden hunger, realizing this potential will require informed investments and complementary capacities from the private, non-profit and public sectors.

## 2 Background

Nutrition in the first 1000 days after conception critically shapes child development. Undernutrition during this period increases the risks of morbidity, mortality and impaired growth, and motor and cognitive development (Dewey and Begum, 2011; Martorell, 1999; Victora et al., 2010). The long-term implications of early-childhood undernutrition can include shorter adult stature, schooling deficits, lower productivity, decreased offspring birthweight, and delayed cognitive development (Hoddinott et al., 2013; Martorell et al., 2010; Victora et al., 2008). Ensuring adequate nutrition for children thus has direct and potent economic development effects (Alderman, 2010; Alderman and Behrman, 2006).

While a food-based approach to improving dietary quality through increased and routine consumption of nutrient-rich foods is generally acknowledged as the preferred long-term solution to undernutrition, strategies that involve fortification and/or supplementation can be implemented in the short-term and help meet high nutrient needs of very young children during the period of complementary feeding from 6 to 24 months of age (Dewey and Vitta, 2012; Vosti et al., 2015). Fortified food blends that have been specifically formulated for young children represent one such option, but concerns about breastmilk displacement, high variability in the amount of the product (and therefore the quantity of nutrients) consumed, and the limited dietary diversity associated with relying on a single food has sparked the development of micronutrient powders and SQ-LNS, both intended for home fortification (Dewey and Vitta, 2012). Compared to micronutrient powders, which contain only micronutrients and are intended to be added to food just prior to consumption, SQ-LNS products embed these micronutrients in a lipid base and deliver energy (∼118 kcal/day’s supply) and protein (2.6 g/day’s supply) along with essential fatty acids and key macrominerals not contained in micronutrient powders (Arimond et al., 2013). Moreover, the fat content of SQ-LNS may enhance the bioavailability and absorption of fat-soluble vitamins (Dewey and Vitta, 2012).

Although SQ-LNS was developed to supplement the diet of target children at a rate of one sachet per day, even partial supplementation may be sufficient to improve child growth and development. Since efficacy trials are typically designed to test the impact of per-protocol supplementation, precise evidence of benefits due to partial supplementation is scarce.^5^ Recent evidence from a different nutrition trial in Western Kenya for similar daily supplements suggests that even a much lower supplementation rate of 0.13 sachet per day on average conferred measurable health benefits (Suchdev et al., 2016).^6^ Given the lingering uncertainty regarding what level of partial SQ-LNS supplementation is sufficient to generate expected benefits, we assess the sensitivity of our results to different sufficiency thresholds below.

While we have learned much in recent decades about key dimensions of child nutrition, important limitations to our understanding persist – limitations that are important as a backdrop to our analysis. Child growth and development are complex processes (Black et al., 2013), and the individual and collective effects of the three general stressors to these processes (undernutrition, infections, and disease) are not completely understood (Hendricks, 2010) and may well be household- or even individual-specific (Dewey and Adu-Afarwuah, 2008).^7^

As background to the study location, Burkina Faso is a low-income country characterized by high rates of early childhood undernutrition and low levels of public and private spending on health.^8^ Of roughly 3 million children under five in Burkina in 2012, 32.9% were stunted (height-for-age z-score <−2), 10.9% were wasted (weight-for-height z-score <−2), and 16.2% were born at low birthweight (<2500 g) (Ministere de la Sante, 2012). Meanwhile, from 2011 to 2015, average annual health expenditures, including both public and private expenditures, were $46 per capita in Burkina Faso, compared to $101 per capita in Sub-Saharan Africa.^9^ Burkina Faso became a member of the Scaling Up Nutrition (SUN) movement in 2011 and has developed a 6-year, US$70.7 m plan to address undernutrition (Scaling Up Nutrition, 2014). Approximately 66% of the plan’s budget was allocated to nutrition-specific interventions, of which complementary feeding of young children in the SQ-LNS target age range (6–24 months) is an integral part.

This study is part of a broader International Lipid-Based Nutrient Supplement (iLiNS-Zinc) nutritional trial^10^ in Burkina Faso, which has found encouraging effects of SQ-LNS on the physical growth and cognitive development of young children (Hess et al., 2015; Prado et al., 2016). The trial was conducted in 34 communities in a large corridor north of Bobo-Dioulasso in the south-western corner of Burkina Faso. The experimental markets described below targeted all established market catchment areas in this corridor as the nutritional trial was winding down. Baseline measures of wasting and stunting from this study are on par with national measures in Burkina Faso and indicate that most young children in this area are at risk of undernutrition that is serious enough to restrict their physical growth (Hess et al., 2015). While there is some evidence that children from poorer households are more vulnerable to wasting, the risk of stunting appears much more uniform across this population. The iLiNS-Zinc nutrition trial, through the provision of SQ-LNS along with monitoring and treatment of malaria and diarrhea, reduced the prevalence of stunting in the study population from 39.3% to 29.3% when the study children were 18 months of age (Hess et al., 2015). To quantify the value of a ten percentage point reduction in stunting in terms of lives saved, we estimate that scaling up this intervention nationwide in Burkina Faso while achieving a similar reduction in the prevalence of stunting among all children under age five would save an average of 2516 lives each year over the next ten years. A national intervention on this scale would avert an average of 69,621 Disability-Adjusted Life Years (DALYs) annually.^11^ This large potential effect is more than 10% of the total disease burden in Burkina Faso due to water, sanitation and hygiene.^12^ Our exploration of the persistence of household demand for SQ-LNS is motivated by these large stakes in conjunction with the fact that free public distribution is not likely to be a viable delivery platform anytime soon.

## 3 Research design and data

**Fig. 1:**
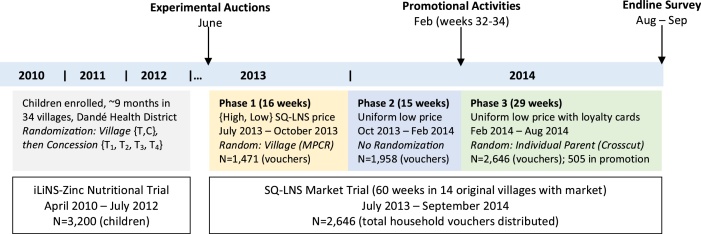
Timeline of research activities and randomization design details of the SQ-LNS market trial relative to the initial iLiNS-Zinc nutritional trial. Our main data source consists of household purchases tracked weekly by voucher booklets. We collected additional data for a subset of these households at the time of the experimental auctions, the promotional activities and during the endline survey.

In the context of the iLiNS-Zinc nutritional trial described above, we designed a series of research activities to evaluate households’ valuation of and demand for SQ-LNS over the short and medium term, distill implications for public policy action regarding retail outlets as a potential delivery platform for these and similar products, and assess the potential for hybrid delivery models that leverage to some degree private demand. As depicted in the research timeline (Fig. 1), the research design for evaluating demand for SQ-LNS built explicitly on the iLiNS-Zinc nutritional trial and was launched one year after this trial was completed.

We used two different approaches to assess rural households’ demand for SQ-LNS. First, we conducted experimental auctions in June 2013 to elicit incentive-compatible experimental WTP in order to provide estimates of initial demand. Second and using these auctions as a point of departure, we then launched a market trial in which we contracted vendors in a subset of the iLiNS-Zinc villages to maintain supplies of, to sell, and to track sales of SQ-LNS (under the local name *Fanga Degue*) for just over one year. Before describing these approaches in detail, we provide an overview of the sampling procedures we used to define the villages and households in our study.

### 3.1 Sampling frame

The approach we devised to draw villages and households into our research sample directly reflected our primary research objective to create and leverage experimental markets for SQ-LNS in our study area to understand household demand. Local retail markets provided the delivery platform for our study, which required that we work in villages with established vendors. Of the original 34 iLiNS-Zinc villages that define our study area, 14 villages had permanent retail shops. We contacted all vendors with permanent retail shops in all of these villages and ultimately engaged most of these vendors (32 in all) as distributors for this project. In all vendor villages, rates stunting and wasting were high by international standards.^13^ Importantly, these 14 villages also host weekly markets that attract people from surrounding villages, including the other 20 iLiNS-Zinc villages, and therefore provide the most promising retail distribution platform in this rural setting.

As for selection of households, we aimed to include in our research sample all households with access to our SQ-LNS vendors that had children in the target age range of 6–24 months. We intentionally minimized direct contact between target households and the research team in order to retain as much of the natural market setting as possible. This design objective led to a relatively open and unstructured sampling frame that used voucher booklets as the primary way to include households in the sample and to track their SQ-LNS purchases. Households could access these SQ-LNS voucher booklets from friends at any time, from vendors upon making their first SQ-LNS purchase, or during promotional activities undertaken during the second half of the market trial period, and thereby enter our sample. While we instructed our vendors and promotional teams to ensure that booklet recipients indeed had a target age child in their household, we used this as more of a guideline and a point of education about the intended usage of SQ-LNS than as a strict criterion for participation.^14^ Through their use (or absence of use) of the booklets, we can track purchases of all households who make at least one purchase. This constitutes our full sample of households in this study.

At the conclusion of the market trial, we randomly sampled a subset of households with voucher booklets – stratified by intensity of demand over the course of the trial – and conducted a detailed endline survey. To construct this sub-sample, we stratified households based on their SQ-LNS purchase patterns, including one strata of households with target-age children that never purchased the product.^15^ All households in our sample that were *not* selected for this endline survey only interacted with our contracted vendors and had little or no direct interaction with our research team.

For more details on the sampling frame and randomization design of the market trial, which is the primary focus in this paper, see the CONSORT diagram provided in supplementary Figure S1.

### 3.2 SQ-LNS demand elicitation via experimental auctions

When assessing demand for a new product or service, it is important to establish a common understanding of and appreciation for the relevant features of the new product or service. In the case of SQ-LNS, this raises two key questions: (1) What specifically should households know and understand about the intended usage and potential benefits of SQ-LNS in order to assess their valuation of the supplement? (2) How should this information be conveyed to rural households, especially in cases of limited caregiver or household head literacy? The broader iLiNS-Zinc project rigorously evaluated the impact of SQ-LNS on various growth and development outcomes of children (Hess et al., 2015). While these results are now available as an evidence base with which to communicate the potential benefits of SQ-LNS, these results were only starting to emerge at the time of the experimental auctions and the launch of the market trial. We worked closely with nutrition collaborators to create the informational scripts to be used in the auctions and to develop packaging, placards, and other promotional materials that were used in the SQ-LNS market trial. Using these communication materials, we informed caregivers of children in the target age range that: a) the average diets of children in the area are deficient in one or more of the micronutrients that nutritionists believe are important for child growth and development, b) the SQ-LNS product described/offered to them contains all of the micronutrients that nutritionists believe are needed for children to grow and develop according to their genetic potential, and c) the SQ-LNS product was designed to fortify children’s food and not replace foods consumed as part of their usual diet. Appendix C describes the information provided to households and how it was conveyed.

As described in detail in Appendix B, we conducted a series of experimental auctions for SQ-LNS in each of the 14 villages in the iLiNS-Zinc study area that would be part of the market trial. The objective of this exercise was to provide bounds on (rather than precise estimates of) household demand for SQ-LNS. The auction sessions, conducted in June 2013, marked the beginning of the market trial and involved fathers and mothers (N = 505) of children in the target-age range, most of whom did not previously participate in the iLiNS-Zinc trial.^16^ Auction participants were given an opportunity to purchase a week’s supply of SQ-LNS for their children using a discretized Becker-DeGroot-Marschak (BDM) mechanism.^17^ Because the efficacy of SQ-LNS depends on regular consumption throughout early childhood, we asked a series of follow-up questions about WTP in the long-term. Specifically, we asked if a participant would pay her maximum WTP for SQ-LNS each week until her child was 24 months. The price was then increased or decreased in small increments until the participant changed her answer.

Data from this incentive-compatible auction offer initial insights into household demand for SQ-LNS (see Appendix B) and, more substantively, provide the point of departure for the price points used in the market trial. Fig. 1 depicts individuals’ valuation of SQ-LNS as demand curves. The solid demand curve in this graph is constructed based on individuals’ incentive-compatible willingness-to-pay (WTP) for a one-week supply of SQ-LNS. The dashed demand curve, labeled ‘(Anchored) Hypothetical Long-Term’, is constructed based on individuals’ stated WTP for SQ-LNS for consistent weekly purchases for the full 18 months of recommended usage beginning when the child is 6 months old. Although partially incentive compatible, this long-term WTP is useful because it was elicited immediately following the auction for a one-week supply and is therefore anchored to the incentive-compatible initial WTP. The comparison of the two curves suggests that persistent demand for SQ-LNS is roughly 40% lower than demand for a single-week supply. For example, whereas 50% of participants were willing to pay $1 or more for a week supply, only 30% claimed to be willing to pay this amount consistently over 18 months of usage. The horizontal solid lines in the figure show the high and low market prices used in the market trial. Because the trial was designed to assess demand, we only considered potential demand and not production costs when setting these prices. Indeed, even the high price was below estimated production costs of approximately US$ 0.14/day or US$ 0.98/week (Lucas et al., 2015). The horizontal dashed lines show average cash expenditures for households in the broader iLiNS-Zinc project: the low price in the trial is roughly equivalent to the average weekly cash expenditures on children per child under age 10 on clothes, sweets, school fees, and toys.^18^ While the low price used in the market trial might be prohibitive for relatively poor households, it is on par with per child spending on snacks and sweets for many households reporting average and above average cash expenditure.

### 3.3 SQ-LNS market trial

Conducted in the same 14 villages that hosted experimental auctions for SQ-LNS and launched immediately following the experimental auctions in each village, the market trial aimed to rigorously evaluate the persistence of demand for SQ-LNS in a naturally-occurring market setting familiar to households in our study area. For this purpose, we engaged one or more local vendors^19^ in each village as collaborators (32 vendors in all, 29 of whom were engaged for the full duration of the trial). We established by contract the terms of the collaboration, including a small commission the vendor would receive for each sachet they sold. Along with an initial inventory of SQ-LNS, each vendor was given a placard that included information about how SQ-LNS is to be consumed and by whom, and its potential benefits.

Each sale was registered using a slip from a voucher booklet; vendors would write the number of sachets sold and the date and put the slip into a lockbox along with the cash paid by the customer. Voucher booklets were distributed to all households who had participated in the iLiNS-Zinc nutrition trial (iLiNS households) and auction participants. Three extra booklets were given to iLiNS households – booklets they were encouraged to distribute to friends. Vendors were also given booklets to keep on hand for interested customers who had not yet received a voucher booklet. Using a unique, household-specific code on each voucher booklet, we are able to track purchases over time. To ensure these purchases were as close to naturally-occurring as possible, we did not collect additional data from purchasing households during the trial. While this imposes some limitations to the analysis (e.g., we cannot track in real-time who specifically consumes purchased SQ-LNS within the household), this design minimizes Hawthorne effects and maximizes external validity in order to shed light on implications for market delivery as it more closely matches any impacts of actual retail delivery of SQ-LNS. Recording weekly household purchases over the full 60 weeks of the trial also enhances statistical power (McKenzie, 2012). Appendix C contains additional detail on how the market trial was implemented.

This market trial was implemented in three separate phases with an initial village-level randomization followed by a subsequent cross-cutting individual-level randomization. The logistical complexities of establishing the necessary relationships with local vendors as the basis for this market trial necessitated this sequential randomization design. In phase one, which was launched in July 2013, we randomly assigned seven of the villages to a low-price treatment and the other seven to a high-price treatment. After 17 weeks, phase two of the trial was marked by reducing all high prices to 150 CFA/strip (one week’s supply for one child) so that a uniform price prevailed across all villages and vendors. Comparing purchases in this phase with those in phase one provides a ‘within-village’ angle on price sensitivity of demand. It also enables us to analyze the dynamics of market demand in these villages by, among other things, contrasting responses on the intensive margin (current clients increasing purchases) and the extensive margin (new clients entering the market).

For improved statistical power during phase 1, we used a ‘matched pair cluster randomized’ (MPCR) approach to assign low and high price treatment status. We paired villages based on observable demand and market characteristics before randomly assigning the low and high initial price within each pair. As detailed in Appendix C, to match villages in this MPCR approach, we exploited baseline census data, market characteristics data, and detailed socio-economic data to construct a factor analytic matching index that was then the basis for paring villages with their nearest neighbor. With the village pairs formed, a simple coin toss determined which of these villages (vendors) would sell SQ-LNS at the low price (150 CFA/seven-sachet strip; $0.30) and which would sell at the high price (300 CFA/seven-sachet strip; $0.60).^20^ Figure S1 includes a map of these 14 villages that indicates the village pairings and the random price assignment.

Finally, in phase three, which was launched in weeks 32–34 of the market trial, we introduced an individual-level, non-price randomization that involved promotional sessions on market day in each of the villages. During the sessions, large groups congregated around the loud speakers and project team as an entertaining member of the project team informed caretakers of target age children about the use, availability and potential benefits of SQ-LNS and then led a question-answer interaction with the crowd. To ensure representation from both male and female caretakers from target households, we actively recruited parents of young children. This was followed by a popular prize wheel for participants who cared for target age children (N = 505 of which 251 male),^21^ often including two members of the same household (e.g., a mother and a father), where each participant had a chance to win a free strip of seven SQ-LNS sachets, a promotional hat and t-shirt, or the hat and shirt plus a product loyalty card that enabled the recipient to redeem 28 empty SQ-LNS sachets for a small reward for a maximum of four rewards.^22^ See Appendix D for additional details about these promotional sessions.

By the end of the trial, over 2600 households had acquired a voucher booklet, 80% of which were used at least once and over half of which were distributed by iLiNS households to their friends and vendors to customers. The average number of sachets purchased per day per voucher booklet is 0.10, but there are clear differences in this average demand across initial high and low price villages (0.13 and 0.31, respectively, in phase one) and between those who did and did not receive the loyalty card in phase three. Detailed summary statistics by phase of the market trial are provided in supplementary Table S1. In the analysis of the next section, we explore these patterns econometrically.

### 3.4 Additional sources of household and other data

In order to characterize household demand for SQ-LNS using the market trial data described above, we need to know something about these households. We collected these data from different households at different times depending on their participation in the study. For those participating in the clinical nutritional trial of the iLiNS-Zinc study – in either a treatment or a control arm – we collected detailed socioeconomic data at the individual, household, and concession levels at enrollment and again at the project endline (when enrolled children completed the treatment regime, at 18 months of age). For auction participants, we collected basic household and individual participant data before or after the auction session. These household data are less detailed than the enrolment and endline data that were collected in the context of nutritional trial, but nonetheless allow us to characterize basic demographic and economic features of these households.

The majority of the households represented in this study, however, did not participate in either the nutritional trial or the auction. Instead, they enter our data by purchasing SQ-LNS using a voucher booklet that they received from a friend who participated in the nutritional trial or directly from one of our vendors. In order to maintain a natural market setting for the trial, we opted not to contact these households for the duration of the trial. Throughout the market trial, therefore, our only link to these households was via their voucher code, which indicated their village of residence and their respective patterns of SQ-LNS purchases, but nothing more. At the conclusion of the study, we randomly selected a subsample of these households, stratified by SQ-LNS purchases, to participate in an endline survey that included several socioeconomic details and questions about their usage and perceptions of SQ-LNS. With these data we are able to include additional demand determinants in the models we estimate below.

### 3.5 Aggregate SQ-LNS demand & measures of demand persistence

**Fig. 2:**
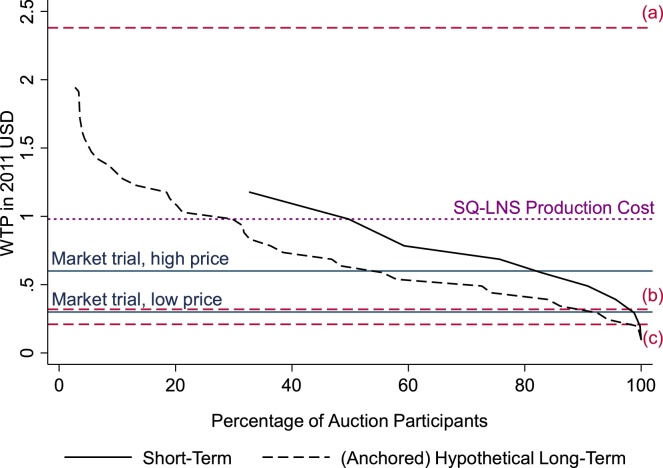
Demand curves for SQ-LNS based on participants’ WTP in experimental auction for a week’s supply and on their (anchored) follow-up anchored-hypothetical long-term WTP. Short-term WTP is truncated at approximately $1.20, the maximum price on the price list used in the auctions. (Anchored) hypothetical long-term WTP > $2 omitted from the figure (1.8% of auction participants). The high and low price levels for the market trial are indicated with solid horizontal lines. The dotted line indicates the estimated cost of producing a week’s supply of SQ-LNS at a new factory in Niger ($0.98; iLiNS Project (2015))^46^. Dashed lines reflect average household expenses from iLiNS baseline surveys: (a) average weekly cash expenditures on food per capita, (b) average weekly cash expenditures on children per child under age 10 (clothes, sweets and snacks, school fees, toys, etc.), and (c) average weekly expenditures on cell phone recharges per capita.

We first provide a few graphical depictions of the evolution of SQ-LNS purchases over the course of the market trial. Fig. 2 shows the evolution of average demand for SQ-LNS by initial price over the course of the 62 week market trial.^23^ Total sales show a steady decline over the trial and converge on about 900 sachets (128 strips) per week during the second half of the trial. The effects of lowering prices in the initial high price villages after week 17 and of the promotion and loyalty card distribution around week 33 are apparent in this figure.

To better interpret total sales by week, we normalize total sachets sold by the estimated number of target age children in the 14 villages included in the trial.^24^ This measure of demand – which provides a rough proxy for ‘community compliance’ – indicates the average number of SQ-LNS sachets purchased in a community for each target child in the community. Recall that full supplementation is one sachet per child per day. This measure is obviously crude because we do not directly observe consumption by children and overstates actual compliance if SQ-LNS is shared among non-target household members, but it provides an indication of the coverage of retail distribution. Table S3 reports the number of target-age children in each village as well as the overall compliance rate (total sachets sold per day / target-age child) for each village in our sample. The overall compliance rate is under 5%, which suggests that 95% of the need implied by full compliance is unmet by the market trial, and falls short of even the lower bound sufficiency threshold of 13% (Suchdev et al., 2016). We compute the ‘coverage rate’ as the number of voucher booklets per target-age child. In most villages, there was well over 50% coverage by the end of the trial, but many of these voucher booklets were never used. When we include only ‘active’ vouchers in this calculation, we have an overall coverage rate of 61%. Taken together with the compliance rate, this suggests that much of gap between recommended and actual SQ-LNS consumption is due to infrequent or inconsistent purchases by target households with voucher booklets in hand.

**Fig. 3:**
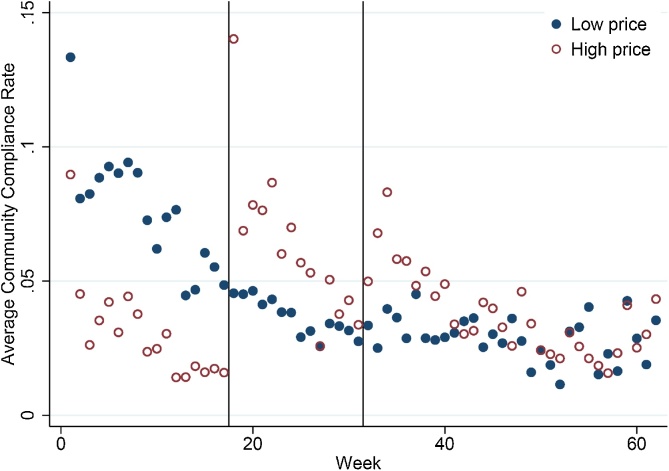
Average community compliance rate by week for villages with initial high and low prices (community compliance rate computed as (total sachets sold per day in village / number of target-age children in village)). Vertical lines indicate the transitions from phase one to two (high price lowered to low price) and from two to three (promotion and individual-level randomization of loyalty card).

Fig. 3 shows the evolution of the weighted-average community compliance rate for villages with the low price throughout the market trial (L-L-L) and those with the high price in phase one (H-L -L). The effect of the initial high price on compliance is clear in this figure, as is the response to the price drop that marked the beginning of phase two. The response to the promotional activities at the beginning of phase three appears to be isolated to villages with initial high prices, suggesting the possibility of path dependent demand. The general decline in the compliance rate over the 62 weeks of the trial that is evident in this figure could have a variety of explanations (e.g., cumulative liquidity constraints that bind over a period of many weeks, fading novelty or attention, etc.), but is not likely due to Hawthorne effects since the households purchasing SQ-LNS during the trial were not directly observed and only interacted with local vendors in a natural market setting.^25^ At this community level, supplementation rates after the introductory low price week are clearly below even the extreme lower bound sufficiency rate of 0.13 sachet per day (Suchdev et al., 2016).

Using the unique voucher codes, we can track purchases of individual households. A few summary statistics from these voucher-level data will help to set the stage for deeper analysis presented below. Since it is formulated as a daily supplement, one of the crucial demand aspects for SQ-LNS is the consistency of demand over time, which we refer to as demand persistence. There are several potential ways to measure demand persistence. We choose to measure it using a three-week moving average of the number of sachets purchased per day.^26^

## 4 Analysis and results

The research design and data described above open several potential perspectives on SQ-LNS demand and demand persistence, and hence on market-based distribution platforms for micronutrient supplements. We begin by estimating the price elasticity of demand using weekly sales by village and the random assignment to low and high initial prices in the market trial. We account for the MPCR structure of the research design and separately estimate the elasticity for initial and repeat purchases in order to shed light on the price sensitivity of one measure of demand persistence. The bulk of this section contains voucher-level analyses that push beyond weekly sales by village and allow us to treat households as the unit of analysis. These voucher-level analyses require careful treatment of non-purchases, which we discuss in detail. We then proceed with analyses of demand persistence, loyalty card effects, and the impact of household characteristics on SQ-LNS demand. Given strong seasonal welfare fluctuations in our rural study area due to heavy reliance on rain-fed agricultural production and incomplete intertemporal smoothing options, we provide complementary and exploratory analysis of demand for SQ-LNS as a function of timing relative to key cropping stages and to rainfall realizations in Appendix G.

### 4.1 Market-level price elasticity of demand for SQ-LNS

In this sub-section, we exploit two parts of the market trial to estimate the price elasticity of demand for SQ-LNS: the stage-one MPCR-based low-high price treatment and the price drop after week 17 that marked the transition to stage two of the trial. In the first case, we estimate (arc) elasticities directly using total sales by village. Since the low price (150 CFA/strip) is 50% lower than the high price (300 CFA/strip), we can directly compute an arc elasticity of demand for SQ-LNS based on the percent change in demand in low price villages relative to high price villages.^27^ We normalize total SQ-LNS demand by the size of the target population, which we estimate as the number of children under age two in each village. Specifically, if we denote the total number of sachets sold in low and high price villages, respectively, as $Q_{L}=\sum_{h}q_{Lh}$ and $Q_{H}=\sum_{h}q_{Hh}$, where *h* indexes households, and their respective target populations as $N_{L}$ and $N_{H}$, then a raw arc elasticity of demand is given by:

(1) $$\eta_{Raw}=\left|\frac{\left(Q_{L}/N_{L}-Q_{H}/N_{H}\right)/\left(Q_{H}/N_{H}\right)}{-50\%}\right|=2\left(Q_{L}/N_{L}-Q_{H}/N_{H}\right)/\left(Q_{H}/N_{H}\right)$$

Note that since we normalize by target population size, this elasticity indicates the price sensitivity of demand for SQ-LNS in terms of sachets per target child.

In order to reflect the MPCR structure of the market trial, we devise a pairwise-corrected arc elasticity of demand based on the MPCR population estimator proposed by Imai et al. (2009). This pairwise-corrected elasticity, essentially a weighted average of the pairwise arc elasticities of demand, is as follows

(2) $$\eta =\sum_{k=1}^{7}\frac{\left(N_{Lk}+N_{Hk}\right)}{N}\frac{2\left(Q^{Lk}/N_{Lk}-Q^{Hk}/N_{Hk}\right)}{Q^{Hk}/N_{Hk}}$$

where subscript *k* denotes the matched village pairs and subscripts *L* and *H* indicate the random assignment of the villages in each pair to low and high price, respectively.

Although villages were assigned to treatment, in a few cases households traveled to neighboring villages to take advantage of lower prices. Our vouchers enable us to pinpoint such ‘displaced’ sales and suggest that they are relatively rare, occur predominantly between two specific high-price villages and two low-price villages, and were virtually non-existent until the second month of the trial. As a correction for these displaced sales, which would artificially inflate our arc elasticity measures, we attribute sales to the home villages of the buyers. This is a conservative correction for displaced sales because it implicitly assumes that households would have purchased SQ-LNS at the high price in their home village if it had not been available in another village at the low price.^28^ We prefer to be conservative in this case because our vouchers do not enable us to test for missing purchases from households that simply choose not to purchase SQ-LNS once they learned of a lower price in another village. While we are confident based on reports from our market agents that such missing purchases are rare, we prefer to be conservative at this stage.

We use bootstrapped samples to generate standard errors on these arc-elasticity estimates (Table 1).^29^ In addition to the pairwise-corrected arc elasticities computed according to Eq. (2), we report the raw overall arc elasticity using six weeks of purchases before and after the price decrease in the initial high price villages. Demand for SQ-LNS is extremely price sensitive. This is particularly true for the repeated purchases required for effective supplementation. By comparison, prior estimates of the arc price elasticity of healthcare demand in rural Burkina Faso are -0.8 overall and -3.64 for children under age one (Sauerborn et al., 1994). This prior estimate for children is similar to our point estimate for first purchases, which seems sensible as most visits to local health clinics for medical attention are one-off visits that are more like first purchases in our context. Repeat purchases impose a recurring cost and are qualitatively different. Prior estimates of healthcare demand elasticities may give some guidance to the price sensitivity of demand for therapeutic healthcare, but they are unlikely to be useful for characterizing demand for preventative investments such as supplements.

**Table 1:** Estimated arc price elasticities of demand for SQ-LNS corrected for pairwise matching of low and high price villages and assuming a 50% price reduction from high to low price.

	Estimate	Bootstrapped Std Error	95% CI
Overall elasticity	−6.0	(0.89)	−7.7,−4.27
Elasticity: first purchases	−4.2	(1.21)	−6.6,−1.8
Elasticity: repeated purchases	−7.7	(1.68)	−11.0,−4.4
Elasticity: within initial high price villages†	−6.0	(0.87)	−7.7,−4.3

† Includes six weeks before and after the 50% price reduction that occurred at week 17.

Normally, such high price elasticity of demand might compel a profit-maximizing firm to lower prices in order to increase revenue, but things are not so simple in this case since the low price in our market experiment is below production costs. Moreover, while the retailer may maximize profits in the classical sense, the SQ-LNS manufacturer faces a complex set of incentives and influences from large multilateral agencies such as WHO and UNICEF. Still, these high price elasticities of demand and the especially high elasticity for repeat purchases provide important insights in this case: Hybrid public-private delivery platforms for products like SQ-LNS might dramatically expand the number of target children reached through efforts to reduce retail prices. Whether such efforts can achieve high levels of persistent supplementation is a question we address in the subsequent voucher-level analyses.

### 4.2 Voucher-level effects of treatment on SQ-LNS demand persistence

Analyzing weekly household-level SQ-LNS purchases enables the estimation of richer models of price sensitivity from phase one of the market trial. Leveraging purchases by households requires assumptions about non-purchases. We assume that once an adult member of a household, typically a parent, receives a voucher, weeks without any SQ-LNS purchases are true zeros and indicate a decision by an eligible household not to purchase the supplement that week.^30^ This assumption is discussed in detail in Appendix F. With this conservative assumption in place, we estimate four voucher-level demand persistence models. First, we estimate overall average effects of the low price treatment (phases 1 and 2) and the loyalty card treatment (phase 3). Next, we estimate a slightly modified specification to generate conditional demand profiles that graphically depict the dynamics of SQ-LNS demand within the three phases of the market trial. Third, we specify habit formation demand models that allow the estimation of treatment effects on both contemporaneous demand and on habit formation. Finally, we estimate a survival model to test the effect of price and non-price treatments on the duration of supplementation above a minimum “sufficient supplementation” threshold.

To estimate the overall average treatment effects for each phase of the trial, we use the following parsimonious demand specifications:

(3) $$D_{ijt}^{MA}=\frac{\sum_{t=t-3}^{t}D_{ijt}}{3}=\alpha_{0}+\alpha_{1}lowprice_{j}+\phi_{t}^{Week}+\phi_{j}^{VillagePair}+\varepsilon$$

(4) $$\begin{array}{c} D_{ijt}^{MA}=\alpha_{0}+\alpha_{1}lowprice_{j}+\alpha_{2}loyaltycard_{j}+\alpha_{3}(lowprice_{j}\times loyaltycard_{j})\\ +\phi_{t}^{Week}+\phi_{j}^{VillagePair}+\varepsilon \end{array}$$

where $D_{ijt}$ indicates SQ-LNS demand measured as the number of sachets purchased per day for household *i* in village *j* and week *t*, $D_{ijt}^{MA}$ is the three-week moving average demand, $lowprice_{j}$ denotes villages with the low price treatment in phase 1, $loyaltycard_{j}$ denotes households that received a loyalty card during the promotion that launched phase 3, $\phi_{t}^{Week}$ is a week fixed effect to allow for common weekly demand adjustments throughout the study area, $\phi_{j}^{VillagePair}$ is a village pair fixed effect to account for the MPCR design, and the disturbance term is clustered by village.^31^ As an alternative to sampling-based inference with clustered standard errors, we use randomization inference (RI) to construct p-values of the treatment coefficients. We use specification (3) to estimate treatment effects for phases 1 and 2, where $\alpha_{1}$ for phase 2 indicates the effect in initial high price villages of a reduction in price to the low price level. We use specification (4) to estimate the treatment effect of receiving the loyalty card while controlling for any path dependence that may emerge from the phase 1 treatment status. We include only households that were represented in the promotional activities in this phase 3 estimation.

Table 2 displays these average treatment effects and shows strong and robust effects of both the low price and loyalty card treatments in their respective phases on SQ-LNS demand. The low price treatment in phase 1 increased demand by 0.18 sachet per day. This 40% increase in demand above the 0.44 sachet per day constant is statistically significant in terms of both clustered standard errors and RI p-values. In phase 2, the price reduction in the initially-high price villages increased demand by 0.052 sachets per day, an effect that is similarly significant. Among households represented in the promotion, the loyalty card treatment in phase 3 induced a 0.10 sachet per day increase in demand, a 63% increase over the lower base of 0.16 sachets per day demand. The steady decline in the estimated constant across these three phases suggests declining demand over the year of the market trial, a pattern we explore more explicitly in the analysis below.

**Table 2:** Average effect of low price treatment in phases 1 and 2 and loyalty card treatment in phase 3 on SQ-LNS demand.

	Phase 1	Phase 2	Phase 3^#^
Constant	0.44***	0.31***	0.16**
	(0.049)	(0.029)	(0.059)
Low price	0.18***	−0.052***	−0.0016
	(0.034)	(0.0095)	(0.015)
	[0.018]	[0.028]	–
Loyalty Card			0.10***
			(0.020)
			[0.000]
Low price × Loyalty Card			0.0012
			(0.040)
			[1.0]
Week FE	YES	YES	YES
Village Pair FE	YES	YES	YES
Observations	20,166	27,445	14,330
R-squared	0.065	0.025	0.055

*** p < 0.01, ** p < 0.05, * p < 0.1. Standard errors clustered by village in parentheses. Randomization Inference-based p-values reported in [].

# Only households represented in the promotion that launched phase 3 are included in the phase 3 estimation.

#### 4.2.1 Low-price treatment effects on SQ-LNS demand persistence

**Fig. 4:**
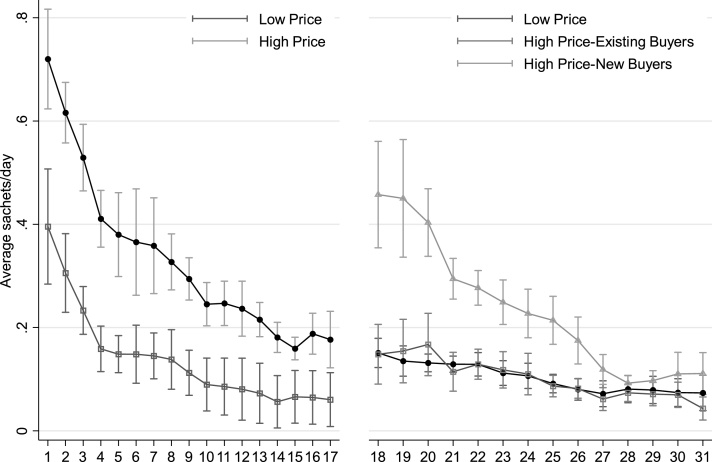
Average persistent demand (three-week moving average sachets per day per household) by week corrected for matched-pair fixed effects during phase one of the trial in high and low price villages (left panel) and after the price drop in high price villages for existing and new buyers in these villages (right panel). Bars depict 90% confidence intervals based on standard errors clustered by village.

To construct conditional demand profiles, we estimate the following simple specification that accounts for the MPCR selection of low- and high-price villages and allows for weekly demand effects of the low price

(5) $$D_{ijt}^{MA}=\alpha_{0}+\alpha_{1}^{\prime }(lowprice_{j}\times week_{t})+\phi_{t}^{Week}+\phi_{j}^{VillagePair}+\varepsilon$$

where $week_{t}$ is a vector of dummies corresponding to week *t*, $\phi_{j}^{VillagePair}$ again accounts for the MPCR design and the disturbance term is clustered by village.^32^ We use this specification to graph the MPCR-corrected average demand across weeks for phase one in low- and high-price villages in the left panel of Fig. 4. Adjusted demand is almost twice as high in low-price villages as in high-price villages. These differences are significantly different across the entire phase, but even in low-price villages demand drops to 0.2 sachets per day on average after two months. The change in persistent demand induced by the price drop after week 17 in initially high-price villages is evident in the right panel of this figure. We distinguish between existing and new buyers in these H-L villages in order to differentiate between intensive and extensive demand changes, respectively. While both types of demand changes are evident in this panel and ultimately result in statistically indistinguishable differences between H-L and L-L villages; new buyers drawn in on this extensive margin have higher demand for SQ-LNS for two months after this price drop. Since this price change was not accompanied by any promotional activities to advertise the new lower price for SQ-LNS, these new buyers learned by word-of-mouth or directly from the vendors that the price had been reduced 50%.

Next, we estimate a habit formation demand model in order to test whether the treatments in this market trial shaped the formation of supplementation habits. In principle, our experimental design allows for tests of both continuous habit formation (i.e., purchases in the recent past shape current purchases) and discrete habit formation^33^ (i.e., purchases for loyalty card holders persist at a higher level even after exhausting the loyalty card rewards). In practice, only five loyalty card winners purchased enough SQ-LNS to receive the maximum four rewards and thereby exhaust their loyalty card, so we cannot test for discrete habit formation. We instead test for continuous habit formation in response to both the low-price and loyalty card treatments by allowing a household’s demand for SQ-LNS to be linked dynamically to their demand in previous few weeks. Specifically, we follow classic habit formation specification in demand analysis (Blanciforti and Green, 1983; Pollak and Wales, 1969) and add lagged SQ-LNS demand as an explanatory variable for current demand.^34^ We allow the low-price treatment to affect both demand levels and habit formation and exploit the panel nature of the data by including Engle-Stone et al

(6) $$\begin{array}{c} D_{ijt}=\beta_{0}+\beta_{1}lowprice_{j}+\beta_{2}D_{ij,t-1}^{MA}+\beta_{3}(lowprice_{j}\times D_{ij,t-1}^{MA})\\ +\phi_{i}^{VoucherSource}+\phi_{t}^{Week}+\phi_{j}^{VillagePair}+(\upsilon_{i}+\varepsilon ) \end{array}$$

where $\phi_{j}^{VoucherSource}$ is a fixed effect for voucher source and $\upsilon_{i}$ is a household random effect.^35^ In the results reported below, we construct RI p-values for coefficients $\beta_{1}$, $\beta_{2}$ and $\beta_{3}$.^36^.

Table 3 shows the results of the continuous habit formation specification estimated for the low-price treatment. The low-price treatment induced households to increase purchases by more than 0.4 sachets per day, an effect that is robust to randomization inference. Based on the coefficient on $D_{ij,t-1}^{MA}$ (average sachets (t-1)), we also see evidence of habit formation. In phase one, the low-price treatment nearly doubled the strength of habit formation from 0.09 to (0.09 + 0.07). In phase two, buyers from phase one, whether in H-L or L-L villages, exhibited stronger habit formation. The habit formation coefficients – but not the interaction with the low-price treatment – is marginally robust to randomization inference. We have estimated (but do not report) this habit formation model for households that were included in the endline survey and for which we therefore have measures of wealth and other observables. While these point estimates suggest that non-poor households are more responsive to the low-price treatment than poor households, these coefficients are imprecisely estimated.^37^

**Table 3:** Results from habit formation demand specification with household random effects.

	Phase 1	Phase 1 & 2	
		All Buyers	Phase 1 Buyers
Constant	0.028	0.28	0.12
	(0.21)	(0.24)	(0.33)
Low price	0.43***	0.41***	0.44***
	(0.13)	(0.13)	(0.13)
	[0.093]	[0.040]	[0.098]
Low price × Phase 2		−0.52***	−0.57***
		(0.12)	(0.15)
		[0.020]	[0.013]
Avg sachets (t-1)	0.090***	0.100***	0.22***
	(0.023)	(0.023)	(0.020)
	[0.089]	[0.24]	[0.15]
…× Low price	0.070**	0.089***	0.046
	(0.032)	(0.031)	(0.031)
	[0.21]	[0.88]	[0.93]
…× Phase 2		0.056	0.19**
		(0.061)	(0.079)
		[0.63]	[0.75]
…× Low price × Phase 2		−0.15**	−0.092
		(0.068)	(0.096)
		[0.78]	[0.91]
Phase 2		−0.035	0.32
		(0.11)	(0.38)
Week FE	YES	YES	YES
Village Pair FE	YES	YES	YES
Voucher Source FE	YES	YES	YES
Observations	18,695	45,653	30,856
Number of voucher	1,442	1,942	1,143

*** p < 0.01, ** p < 0.05, * p < 0.1. Standard errors clustered by village in parentheses. Randomization Inference-based p-values reported in [].

Our third modeling approach addresses a limitation of the habit formation model and provides a complementary angle on supplementation demand by focusing explicitly on the persistence of supplementation. While we find evidence of habit formation, especially in L-L villages, this need not imply that supplementation habits are sufficient to provide the intended physiological and cognitive benefits. Survival models can explicitly account for such a sufficiency threshold. As discussed above, there is little evidence to suggest a clear sufficient supplementation threshold in the case of SQ-LNS, but it is almost surely less than the full 1.0 sachet per day level. We take a lower bound sufficiency threshold of 0.13 sachets per day (Suchdev et al., 2016) as this minimum threshold and define survival as the number of weeks a given household maintains $D_{ijt}^{MA}$ above this level.^38^ Since this supplementation sufficiency assumption is a key assumption in these survival models, we display results for a full range of possible sufficiency thresholds below. Specifically, we estimate the following Weibull hazard model

(7) $$\ln\left[h(t_{ij},lowprice_{j})\right]=\ln\left[h_{0}(t_{ij})\right]+\gamma lowprice_{j}+\phi_{i}^{VoucherSource}+\phi_{j}^{VillagePair}+\varepsilon$$

where $t_{ij}$ is the duration of sufficient supplementation, the error is clustered by village-month, and γ indicates the differential hazard rate – that is, the change in the risk of falling below the 0.13 supplementation threshold – in L-L villages relative to H-L villages. Since households in our market trial area can purchase SQ-LNS at any point, we allow for multiple failures when estimating this model (i.e., households can revive sufficient supplementation and reset their duration after failing to purchase SQ-LNS for several weeks or months).

Table 4 suggests that exposure to the random low price in phase 1 significantly reduces the risk of failure (-0.12). This effect is comparable for households in H-L villages after the price dropped to the low price in these locations, which experienced a reduction in the risk of failure relative to L-L households of -0.15. When we estimate these effects only for the endline survey sub-sample (unreported results), we find no significant wealth-differentiated effects of the low-price treatment on survival.

**Table 4:** Survival model (Eq. (5)) results testing the effect of the low price treatment in phase one of market trial using a sufficient supplementation threshold of 0.13 sachets per day (Suchdev et al., 2016) and allowing for multiple supplementation “failures” for each household.

	Phase 1	Phases 1 & 2
Constant	−2.32***	−1.74***
	(0.12)	(0.034)
Low price village	−0.12***	−0.14***
	(0.025)	(0.026)
	[0.033]	[0.01]
Phase 2		−0.33***
		(0.028)
Low price village × Phase 2		0.15***
		(0.034)
		[0.01]
Pair FE	YES	YES
Voucher Source FE	YES	YES
Observations	13,027	35,240

*** p < 0.01, ** p < 0.05, * p < 0.1. Standard errors clustered by village in parentheses. Randomization Inference-based p-values reported in [].

#### 4.2.2 Loyalty card treatment effects on SQ-LNS demand persistence

We next analyze the effects of the non-price promotion introduced in phase three of the market trial. We are particularly interested in the effect the loyalty card has on purchases and on habit formation. As a secondary objective, we also test whether male and female winners at the promotion affect differently their household’s subsequent SQ-LNS demand.^39^ Regression analysis of our experimental auction data suggested men have a substantially higher long-term WTP for SQ-LNS than women (see Table A2). Further, during the first phase of the trial, several of our vendors shared their perception that the engagement and commitment of the father of the target child^40^ most influenced a household’s purchase patterns: Only households in which the father was committed to the care of his children and saw SQ-LNS as a potentially valuable product regularly purchased the product. The design of the promotional activities enables us to test whether the participation of the father in the promotional sessions affects subsequent demand persistence.^41^

**Fig. 5:**
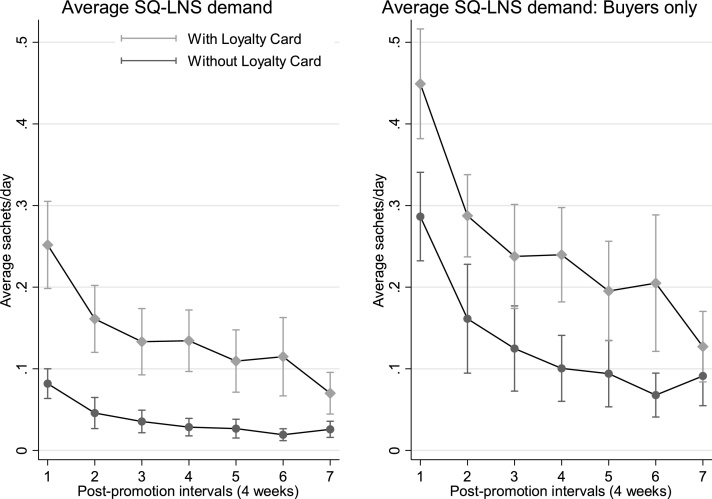
Conditional three week moving average of sachets purchased per day after promotional activities for households that won a loyalty card and those without a loyalty card, including all households that participated in the promotion (left) and only those from the promotion that purchased at least one sachet of SQ-LNS after the promotion (right). Error bars depict 90% confidence intervals based on standard errors clustered by village.

Following the structure of sub-section 4.2.1, we begin with a graphical depiction of the effect of the loyalty card on $D_{ijt}^{MA}$ using a modified version of specification (3) that replaces week dummies with four week interval dummies to fit the loyalty card that was designed to reward four weeks of purchases up to four times.^42^ Fig. 5 displays the resulting conditional demand for SQ-LNS for different four-week post-promotion intervals. The effect of the loyalty card is clear in this figure: The loyalty card induced the average household to purchase two or three times more SQ-LNS for most of phase three. These effects are amplified when we restrict the analysis to households attending the promotion that made at least one purchase during phase three (right panel).

Next, we test the impact of the individually-randomized rewards from the promotion that marked the beginning of phase three using a modified version of the habit formation specification in (4), which continues to include random effects, village clustered errors and RI p-values for treatment coefficients. We replace $lowprice_{j}$ with a set of dummies characterizing the prize(s) a given household received at the promotion.^43^ In column one of Table 5 (all households in promotion; phase 3 only), the effect of the loyalty card on demand in the first period is large and statistically significant. The point estimates for interactions with subsequent post-promotion periods are mostly negative and increasing in magnitude, suggesting that loyalty card effects fade over time. While these fading treatment effects are statistically significant based on the village-clustered standard errors, only the interaction with post-period 7 is significant based on the RI p-values, which provides mixed statistical evidence on the persistence of the demand effect of the loyalty card. As mentioned above, only a few households exhaust the rewards offered by the loyalty card, so these coefficients on post-promotion periods cannot provide evidence on discrete habit formation. We instead evaluate the strength of continuous habit formation as in phase 1. Compared to the phase 1 results in Table 3, we see yet stronger evidence of habit formation during phase 3, which may reflect the selectivity of the sample of households included in this analysis (only those attending the promotion are included in this analysis). We do not, however, see any evidence that the loyalty card amplifies habit formation as the effect of the interaction between lagged demand and loyalty card is statistically zero. The other columns of this table suggest that the loyalty card had stronger demand effects among households in H-L-L villages, although the confidence intervals of these effects overlap. We see statistically stronger differences between households who first received a voucher booklet at the promotion and pre-existing buyers, with the latter responding more strongly to the loyalty card than the former. Although we expected to see the loyalty card having a more pronounced effect on demand when the male received the card in his name, we see no strong evidence in support of this hypothesis. Unreported results with the endline sub-sample hint that non-poor households with a male (but not a female) loyalty card holder may increase their demand for SQ-LNS.

**Table 5:** Habit formation model with buyer random effects for households that participated in promotional sessions. The ‘pre-post promotion’ column includes only households that had purchased SQ-LNS before participating in the promotional sessions that launched phase 3 and uses purchases before and after the promotion to estimate the loyalty card effect on demand.

	Phase 3 Only				Phase 2 & 3
	All	Phase 1 price		New buyers	Pre-existing buyers
		Low	High		
Constant	2.45	4.91**	−1.95	−1.14	2.16**
	(1.83)	(2.21)	(2.47)×	(2.16)	(0.96)
Loyalty card	0.91***	0.78***	1.06***	0.60**	0.30
	(0.18)	(0.15)	(0.37)	(0.25)	(0.30)
	[0.015]	[0.11]	[0.0435]	[0.12]	[0.41]
Avg sachets (t-1)	0.36***	0.36***	0.36***	0.29***	0.38***
	(0.032)	(0.036)	(0.069)	(0.034)	(0.043)
Loyalty card × Avg sachets (t-1)	−0.0090	−0.025	−0.0097	0.011	0.044
	(0.044)	(0.041)	(0.089)	(0.044)	(0.091)
	[0.96]	[0.91]	[0.96]	[0.96]	[0.86]
Post-promo					0.48**
					(0.19)
Loyalty card × Post-promo					1.12***
					(0.36)
					[0.23]
…× Post-period 2	−0.49**	−0.39***	−0.60*	−0.13	−1.03**
	(0.20)	(0.13)	(0.35)	(0.19)	(0.50)
	[0.29]	[0.53]	[0.39]	[0.77]	[0.34]
…× Post-period 3	−0.41*	−0.12	−0.70***	−0.17	−0.66*
	(0.22)	(0.33)	(0.21)	(0.30)	(0.34)
	[0.40]	[0.86]	[0.23]	[0.67]	[0.57]
…× Post-period 4	−0.58**	−0.28	−0.91**	−0.28	−0.96*
	(0.26)	(0.18)	(0.45)	(0.22)	(0.53)
	[0.20]	[0.61]	[0.14]	[0.52]	[0.35]
…× Post-period 5	−0.56***	−0.45**	−0.68***	−0.28	−0.91**
	(0.15)	(0.18)	(0.22)	(0.23)	(0.36)
	[0.18]	[0.43]	[0.28]	[0.53]	[0.39]
…× Post-period 6	−0.38	0.072	−0.85***	−0.016	−0.89**
	(0.28)	(0.34)	(0.28)	(0.38)	(0.44)
	[0.35]	[0.90]	[0.14]	[0.98]	[0.37]
…× Post-period 7	−0.83***	−0.77***	−0.91***	−0.38*	−1.48***
	(0.15)	(0.14)	(0.28)	(0.21)	(0.33)
	[0.048]	[0.17]	[0.12]	[0.36]	[0.13]
Male won loyalty card	0.012	0.036	0.019	−0.14**	0.43
	(0.12)	(0.12)	(0.21)	(0.065)	(0.26)
	[0.98]	[0.97]	[0.98]	[0.75]	[0.82]
Village FE	YES	YES	YES	YES	YES
Observations	13,994	7,683	6,311	9,672	6,349
Number of voucher	481	263	218	336	145

*** p < 0.01, ** p < 0.05, * p < 0.1. Robust standard errors clustered by village in parentheses. Randomization Inference-based p-values reported in []. Unreported controls include week and week squared and dummies for post-promo periods, other promotion prices, couple at promotion, and male at promotion.

We similarly modify the survival specification in (5) by replacing $lowprice_{j}$ with a set of variables characterizing the promotional prizes received by a given household. The results shown in Table 6 illustrate a large and significant effect of the loyalty card on supplementation survival. As before, these effects tend to fade over the course of the post-promotion intervals, although these effects are statistically more precise based on both standard errors and RI p-values. Interestingly, the loyalty card effects for those in L-L-L villages persist six periods whereas the effects for households in H-L -L villages only persist two periods, potentially suggesting a ‘sticky framing’ effect. Estimation results with the endline sample again hint that non-poor households with male loyalty card holders ‘survive’ longer than the same households with female card holders.

**Table 6:** Survival model results testing the effect of promotional prizes of phase three using a sufficient supplementation threshold of 0.13 sachets per day (Suchdev et al., 2016) and allowing for multiple supplementation “failures” for each household.

	Phase 3				Pre-Existing Vouchers	
		Phase 1 price		New Vouchers		
	All	Low	High		Phase 3	Phases 2 & 3
Constant	−1.33***	−1.28***	−1.40***	−2.91***	−1.17***	−1.42***
	(0.11)	(0.098)	(0.17)	(0.35)	(0.21)	(0.14)
Loyalty card	−0.40***	−0.31***	−0.53***	−0.22	−0.38***	0.075
	(0.12)	(0.12)	(0.16)	(0.40)	(0.11)	(0.074)
	[0.013]	[0.11]	[0.033]	[0.77]	[0.013]	[0.33]
Post-promo						−0.062
						(0.038)
Loyalty card × Post-promo						−0.42***
						(0.11)
						[0.005]
… × Post-period 2	0.16	0.041	0.30	−0.12	0.11	0.12
	(0.12)	(0.20)	(0.20)	(0.29)	(0.10)	(0.10)
	[0.39]	[0.86]	[0.33]	[0.87]	[0.58]	[0.53]
… × Post-period 3	0.22	−0.065	0.51***	0.12	0.048	0.051
	(0.14)	(0.20)	(0.14)	(0.35)	(0.091)	(0.090)
	[0.21]	[0.78]	[0.07]	[0.89]	[0.78]	[0.79]
… × Post-period 4	0.21	0.0050	0.42**	0.12	0.026	0.028
	(0.14)	(0.15)	(0.21)	(0.30)	(0.13)	(0.13)
	[0.21]	[0.99]	[0.11]	[0.89]	[0.90]	[0.86]
… × Post-period 5	0.33***	0.15	0.54***	0.27	0.11	0.11
	(0.12)	(0.12)	(0.16)	(0.31)	(0.098)	(0.097)
	[0.04]	[0.48]	[0.05]	[0.71]	[0.56]	[0.49]
… × Post-period 6	0.32***	0.18	0.49***	0.23	0.15	0.16
	(0.12)	(0.12)	(0.16)	(0.30)	(0.098)	(0.098)
	[0.055]	[0.39]	[0.07]	[0.77]	[0.40]	[0.32]
… × Post-period 7	0.38***	0.23**	0.56***	0.20	0.31***	0.31***
	(0.12)	(0.12)	(0.19)	(0.32)	(0.092)	(0.092)
	[0.02]	[0.23]	[0.03]	[0.77]	[0.065]	[0.045]
Male won loyalty card	−0.090	−0.10	−0.059	−0.14	−0.098	−0.15
	(0.085)	(0.075)	(0.12)	(0.28)	(0.13)	(0.12)
	[0.99]	[0.99]	[0.78]	[0.67]	[0.93]	[0.99]
Village FE	YES	YES	YES	YES	YES	YES
Observations	6,556	3,402	3,154	2,689	3,867	5,607

*** p < 0.01, ** p < 0.05, * p < 0.1. Standard errors clustered by village in parentheses. Randomization Inference-based p-values reported in []. Unreported controls include dummies for post-promo periods, other promotion prices, couple at promotion, and male at promotion.

As a quick comparison of the phase-one and phase-three results, consider the effect of the loyalty card relative to the phase one low price. While these were not designed as independent and simultaneous randomization arms, we can nonetheless compare their effects because the low price and the loyalty card (taking into account the value of the rewards offered) both effectively reduced price by 50%. As the primary difference, the loyalty card offered an *ex post* reward after four weeks of consistent purchases instead of the contemporaneous price discount.^44^ Based on the point estimates in Table 3, Table 5, the low price in phase one increased demand by 0.43 sachets per day whereas the loyalty card in phase three increased demand by more than twice as much (0.91). Comparing Table 4, Table 6, the low price reduced the risk of supplementation failure by 0.12 whereas the loyalty card reduced this risk by 0.40. While we hesitate to make too much of this difference given obvious complications (e.g., phases one and three occurred at a different time of year and included a potentially different set of households), this pattern is consistent with the loyalty card inducing a non-price effect on demand that is at least as important as the direct price effect implied by value of the reward it offers, which suggests a potential behavioral mechanism in play.

**Fig. 6:**
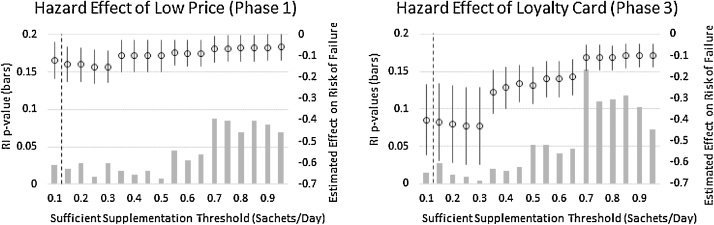
Sensitivity analysis of main treatment effects of low price (phase 1) and loyalty card (phase 3) with respect to the sufficient supplementation threshold (right axis). Randomization Inference (RI) p-values for each treatment effect estimate are depicted as bars (left axis). The vertical dashed lined indicates the lower bound threshold of 0.13 sachets per day (Suchdev et al., 2016) used to generate the hazard model results reported in the tables.

The survival model results reported thus far all use the lower bound sufficiency threshold of 0.13 sachets per day. As mentioned earlier, many consider a higher threshold of 0.5 sachets per day to be more realistic. In the absence of clear evidence, we estimate these models for a range of possible sufficiency thresholds and report the main hazard model effects for the low-price and loyalty card treatments in Fig. 6. While the point estimates of these effects do fade as the threshold approaches full compliance, the basic results at thresholds of 0.13 and 0.5 are quite consistent. For a wide range of sufficiency thresholds, the effect of the loyalty card on survival is far greater than that of a price reduction of an approximately equivalent value.

### 4.3 Household characteristics and SQ-LNS demand

The voucher-level analyses presented thus far use measures of weekly demand constructed from weekly SQ-LNS purchases. For our full sample of households, we only observe how they received and how they used their voucher booklet, which prevents any analysis of how household characteristics shape their SQ-LNS demand. Using the endline sub-sample, we can conduct such an analysis – albeit with less statistical power given the smaller sample.

As shown in supplemental Table S2, we find that household assets – a proxy for wealth – are strongly related to overall SQ-LNS demand, with relatively richer households purchasing SQ-LNS more consistently. We also find that households that purchase more than half of the food they consume, which in our study context is an indication of both liquidity and frequent transactions in local markets, have higher demand persistence. As a test of whether father involvement influences demand persistence, as reported by many vendors, we construct a factor analytic index based on father involvement in SQ-LNS purchase and medical expense decisions. We find that higher father involvement increases a household’s demand persistence, although these estimates are mostly imprecise. In the final column, households that purchased seven sachets or less are not necessarily poorer or richer than the others. Instead, they tend to be more food secure (negative coefficient on hunger score) and have less involved fathers, although this latter coefficient is not significant at conventional levels (p-value = 0.15).

To put these results in context, it is important to reiterate that the market experiment was not designed to test nutritional and growth outcomes associated with households’ market purchases of SQ-LNS. Thus, while the full nutritional trial that preceded it collected complete anthropometric data and took blood samples, the market experiment did not. As a result, we are unable to test whether households’ self-selection into the SQ-LNS market leads to at-risk children receiving supplements at all, and if so, what the effects might have been. Note, however, that in the rural Burkina Faso setting of this study the prevalence of hidden hunger among young children is high enough that targeting is a lower priority than coverage.^45^

## 5 Conclusions

Reducing undernutrition, especially among young children, is the focus of much international development research and policy action. Given the breadth of micronutrient deficiencies faced by these children and diets that are characterized by limited nutrient-rich fruits and vegetables, a general absence of animal products, and periods of food shortages, researchers and policy-makers alike have looked beyond locally available foods to address undernutrition problems. While SQ-LNS products combined with general health monitoring and treatment services have shown promise in our study area and could considerably reduce the burden of disease associated with stunting if access to and usage of these supplements were scaled up nationwide in Burkina Faso moving from therapeutic to preventative formulations raises several important distribution and delivery issues. This paper aims to shed light on these issues and on possible delivery strategies, by improving our understanding of the determinants, dynamics and persistence of household demand for SQ-LNS.

The market trial we conducted sold over 70,000 SQ-LNS sachets during a 60-week period. When compared to the number of target-age children in this study area, this level of sales falls well short of per-protocol supplementation. Specifically, this level of aggregate sales would satisfy the recommended ‘one sachet per child per day’ for roughly 5% of target-age children. While very few households stuck to the per-protocol regimen, over 60% of the households to which these children belong purchased at least some SQ-LNS at some point over the 60-week period. This household demand is very sensitive to price; demand for repeat purchases is especially price elastic. We also find evidence that several non-price factors also shape demand. Promotional activities at the beginning of phase three of the trial revived demand for a period of time. In particular, a simple loyalty card more than doubled household SQ-LNS purchases to levels of supplementation that may be sufficient to confer some health benefits (Suchdev et al., 2016), and had a larger effect on demand than a direct price discount of roughly equivalent value. These or similar promotional activities clearly entail costs, but our research design does not allow us to formally estimate the cost-effectiveness of such a campaign.

Viewed through an economics lens, these results are not surprising. SQ-LNS products are “credence” goods in the sense that benefits that are generally not detectable to caregivers over reasonable time horizons. In this sense, these products are more similar to education than to bed nets. This, combined with the poverty experienced by many households in rural Burkina Faso, makes it unrealistic to expect broad-based compliance with a daily supplementation regimen that requires very frequent product procurement by households. We do, however, see evidence that a minority of less-poor households purchase SQ-LNS at a frequency that may be consistent with sufficient supplementation, suggesting that retail delivery platforms may be viable for *some* households in our study area. Poorer households, however, purchase only limited quantities even at lower prices or with specific promotion, and therefore would not adequately be reached by SQ-LNS products provided through retail markets. The majority of households may only achieve sufficient supplementation with hybrid platforms that involve external subsidies and support. While more research is needed, the effects we find of observable characteristics on demand dynamics provide a point of departure for exploring possible spatial, temporal, or other targeting strategies.

Viewed through a nutritional lens, these results are disappointing because they suggest that these perhaps promising products will require sustained public sector support and may therefore raise difficult tradeoffs with other public investments in nutrition and health. Even in settings (such as ours) where SQ-LNS products have been demonstrated to be efficacious in promoting child growth and development and have the potential to save nearly 7000 DALYs annually among young children and hence may be *socially* cost-effective as a nutritional investment, *private* demand will likely cover only a small part of the production and distribution costs. The fact that SQ-LNS products do not appear to be efficacious in all settings, potentially due to differences in disease pressures, gut infections, and other environmental factors that influence the bioavailability of SQ-LNS, introduces uncertainty at the population and household levels regarding expected growth and developmental benefits. This nutritional complexity underscores the important distinction between nutritional efficacy and the cost-effectiveness of alternative delivery platforms, both of which may hinge on features of the local context.

How might one resolve this impasse between demonstrated efficacy and fundamental delivery dilemmas? Several options are worth considering. First, it may be possible to reduce the dosage of SQ-LNS that young children are recommended to receive; given the high proportion of production and product transportation costs in total costs, doing so would substantially reduce the overall cost of providing SQ-LNS. However, very little is known about the effects on child growth or development of reducing dosages below those used in field-based nutrition trials. Second, per-sachet costs could be reduced. This could be achieved by dramatically increasing the scale of production of SQ-LNS; perennial issues related to product safety might also be more efficiently addressed at larger scale. Achieving such scale economies would almost certainly lead to dramatic changes in existing, or planned, supply chains for SQ-LNS products; small countries, in particular, may be hard-pressed to cost-effectively respond to pressures to establish and maintain large-scale national SQ-LNS production capacity. Third, it may be possible to target at-risk geographic areas and/or seasons for SQ-LNS distribution; information on dietary intake of children could be used to guide such targeting efforts. Finally, hybrid private-public delivery strategies that leverage cost-sharing and comparative advantage merit serious consideration. For example, community-based healthcare personnel could maintain stocks of SQ-LNS products to distribute to target-age children and collect a nominal fee from caregivers.

More generally, the public sector has a clear role to play in providing the investments, infrastructure, and legal frameworks required to enable the private sector to engage more intensively and effectively in these markets. While direct subsidization – a prototypical feature of public-private models – may at least initially play an important role, the public sector can also help reduce production costs by reducing trade barriers and tariffs for key production inputs, or for finished products produced elsewhere. Public-sector procurers of SQ-LNS products also have a fundamental role to play in reducing the uncertainty associated with demand for these products – credible, long-term contracts will be required to stimulate private sector investments. Private firms in the food and beverage sector, and other low-margin/high-volume consumer goods, have proven to be remarkably nimble and innovative in rural African settings. Bringing some of this supply chain expertise to bear on SQ-LNS markets could stimulate crucial innovations in production processes, and in distribution and marketing. But fully tapping this expertise will require the current public and private stakeholders in childhood nutrition to embrace this as an opportunity to build partnerships based on trust, and to ensure that associated regulatory systems function effectively and efficiently.

## Footnotes

46 When the facility producing SQ-LNS is operating at full capacity, the cost of producing a week’s supply may fall to as low as $0.77 (iLiNS Project, 2015),

1 This triggered a massive budgetary and programmatic shift towards therapeutic LNS products by the three major childhood nutrition players: United Nations Children's Fund (UNICEF), Médecins Sans Frontières (MSF) and the World Food Programme (WFP).

2 In contrast to bednets to prevent malaria, it is more difficult for parents to learn about the benefits of micronutrient supplementation. Consequently, short-run subsidies are unlikely to fuel learning in quite the same way (Dupas, 2014), which raises additional challenges to tapping the demonstrated potential of SQ-LNS products to remedy hidden hunger and improve child growth outcomes.

3 We focus primarily on the procurement decision, but acknowledge that proper usage of a preventative health product also entails costs in the form of time and effort. These usages costs clearly exist for durable as well as consumable products. Also, note that while many consumable health products are nonperishable and can be stored, which reduces the required frequency of procurement, liquidity constraints often prevent households from making bulk purchases. Consequently, perishability of preventative products often does not significantly shape procurement frequency for cash-strapped households.

4 The evidence to date on the sustainability of market-based distribution of micronutrients is limited to two studies that monitored the sales of micronutrient powders (MNP) for daily home fortification of complementary foods, one in Western Kenya (Suchdev et al., 2012, 2010; Suchdev et al., 2013) and another in Vietnam (Nguyen et al., 2016). Both of these studies found a high percentage of children who consumed the MNP at least once, but consumption was infrequent, averaging .9 sachets per child per week in Kenya while just 11.5% of children in Vietnam consumed the MNP at least three times per week. Expanding on this work, our research design enables us to track all purchases of SQ-LNS, by customer, for over one year and provides a robust evidence base for evaluating demand persistence, including sensitivity to price and promotional activities, for a nutritional supplement delivered via a market platform.

5 Some practitioners consider 0.5 sachet per day as sufficient for impact, but in the absence of clear evidence this rule of thumb seems to reflect a convenient “every other day” anchor as much as any substantive reasoning.

6 This analysis focused on micronutrient powders rather than SQ-LNS, but the micronutrient content and intended effects are very similar. The health benefits of target children included 60% fewer hospitalizations for diarrhea and 70% fewer hospitalizations for fever.

7 Many key issues related to undernutrition remain uncertain or unknown: definitions of undernutrition are hard to pin down even for basic nutrients (e.g. vitamin A), the effects of shortfalls of particular nutrients are hard to predict even in controlled settings, the effects of shortfalls of collections of nutrients are essentially unknown, and the detailed nutritional status of children in developing countries is rarely known (for exceptions, see Engle-Stone et al., 2015, Engle-Stone et al., 2012). For these and other reasons, SQ-LNS products do not always achieve the expected results (e.g., Maleta et al. (2015)). Although we do not address these nutrition and child development complexities in this paper, they are broadly relevant to our focus on household demand for SQ-LNS and may have implications for delivery and effective use of supplements to prevent child undernutrition.

8 The World Bank estimates the 2014 Gross National Income per capita for Burkina Faso was $710, less than half the 2014 average of $1699 per capita for developing countries in Sub-Saharan Africa. Sources: http://data.worldbank.org/country/burkina-faso and http://data.worldbank.org/region/SSA.

9 Sources: http://data.worldbank.org/country/burkina-faso and http://data.worldbank.org/region/SSA.

10 For details, visit the iLiNS website: http://www.ilins.org/. As described in Fig. 1, these villages were randomly assigned to treatment or control. In treatment villages, “concessions” (large households that encompass extended families) were randomized into four different treatment arms. Other features and details about this clinical nutritional trial are provided in Appendix A and are available in (Hess et al., 2015).

11 Lives saved were estimated from 2019-2028 using the Lives Saved Tool (LiST) (http://livessavedtool.org/) assuming the rate of stunting among all children under five years of age was 39.3% in 2018 and then fell to 29.3% in all subsequent years (2019-2028). We acknowledge that this assumption, which is necessitated in this exercise by the age groupings currently available in the LiST tool, is optimistic given that the observed effect on stunting rates was measured at 18 months of age, and reductions in the prevalence of stunting among children older than two or three years of age are less common (http://thousanddays.org/the-issue/stunting/). DALYs were calculated based on the following assumptions: lives are saved at 18 months of age; life expectancy is 60.5 years (http://www.who.int/countries/bfa/en/); years of life saved are discounted at 3%.

12 Estimating the cost-effectiveness associated with this kind of scaled up intervention is beyond the scope of this analysis, so we stop at estimating these projected benefits of a hypothetical national campaign. For details on the disease burden of water, sanitation and hygiene, see http://www.who.int/quantifying_ehimpacts/national/countryprofile/burkinafaso.pdf (Accessed 11 June 2018).

13 There is some weak evidence in the data from the broader project that these rates are slightly lower than in the more remote villages without permanent retail shops, but these differences are small compared to the disparity with international standards.

14 There is no evidence of SQ-LNS impact – positive or negative – on individuals outside the target age range. Where possible through our collaborators in the field, we reiterated proper usage and the target age range in order to discourage households with little or no expected SQ-LNS benefits from making purchases.

15 See Appendix ED for details of these endline sampling procedures. As a final data layer, we use the location of the villages in our study to merge weekly rainfall experienced during the approximately one-year market trial, by village, into the dataset. These rainfall data, described in greater detail below, are constructed as part of the Famine Early Warning Systems Network (FEWS-NET) of the U.S. Agency for International Development (USAID). Combined with the spatial and temporal variation in SQ-LNS demand, these rainfall data allow us to test for the effects of one key aspect of weather on demand, an important consideration given the heavy reliance on rainfed agricultural production in our study area. This analysis is reported in Appendix G.

16 In principle, households that participated in both the iLiNS-Zinc trial and this SQ-LNS auction were comparable to other auction participants since for both types of households had very young children. In practice, however, the iLiNS-Zinc households had first-hand experience with SQ-LNS and may have been sensitized to nutritional information through their participation in the trial. Moreover, these iLiNS-Zinc households have slightly older young children on average by construction as they have at least one child who recently aged out of the SQ-LNS target age range, which marked the end of their participation in the iLiNS-Zinc study.

17 The BDM mechanism is a standard way to elicit individuals’ valuation of a good or service in experimental auctions. In principle, it provide “incentive compatible” measures of willingness-to-pay in the sense that respondents have no incentive to misreport or misrepresent their true valuation for the good or service. We discretize this mechanism by offering participants a sequence of prices and asking them to decide if they would be willing to purchase SQ-LNS at each price. See Appendix B for additional details.

18 We construct this set of cash expenses based on common purchases that are earmarked for young children. School fees constitute roughly 25% of these children expenses on average. Since SQ-LNS is designed to supplement rather than to replace any food consumed by young children, there is no other compelling benchmark against which to compare demand for SQ-LNS. Enriched milk powder for young children (Nestle’s NIDO®) may be misunderstood by some parents as indistinguishable from SQ-LNS. In these retail shops, the cost of a week supply was close to our high price of 300 CFA, but few households in the full iLiNS-Zinc sample or our endline sample regularly purchased this product.

19 We explored the option of using other outlets in addition to (or even instead of) local vendors. For example, health clinics and pharmacies might make sense as outlets for SQ-LNS. We settled on engaging only local vendors as this would ensure the most widespread access to SQ-LNS, as every village involved in the study has at least one permanent shop, and also emphasized SQ-LNS as a preventative rather than a treatment product.

20 Based on production technologies and input prices in neighboring Niger at the time of the study, even this ‘high’ price did not cover all production costs or the likely mark-up required by vendors. Cost-cutting innovations may help to close this gap, but is unlikely to close it entirely, which underscores the need for some level of public support or subsidization to ensure sufficient consumption of SQ-LNS to generate the expected growth/development benefits, if broad-based provision/promotion of SQ-LNS products becomes part of nutrition/health programs in rural Burkina Faso.

21 Only individuals who cared for children in the target age window were eligibility to spin the prize wheel.

22 Reward options included familiar items in local shops such as small sachets of tea and laundry detergent and sugar (0.5 kg). The market value of these items was approximately 250-300CFA.

23 We observe the date of all purchases. Because most of these purchases involve seven sachet strips (see Figure S2S1), we aggregate purchases into weekly time steps. As a comparison to the average demand in Fig. 2, Figure S3 shows total sachets purchased across all villages included in the market trial.

24 Estimates of the sizes of the target population were based on local census data collected in 2013 by the Centre médicale de Dandé (CMA), Burkina Faso.

25 The only minor departure from this natural vendor interaction was the use of the voucher booklet, which might have distorted purchase decisions but cannot explain the general decline in demand as the booklet was required to purchase SQ-LNS across all 62 weeks of the trial.

26 For an alternative measure, see Figure S5, which is based on the number of weeks a given household purchases at least 7 sachets (i.e., the number of weeks at full compliance). We prefer the three-week moving average as a continuous measure. While price appears to have a significant effect on demand persistence, most households with access to SQ-LNS at the low price still consume well below full supplementation rates. Specifically, while less than 5% of households facing the high price purchase at least one sachet a day, roughly 20% of households in low price villages purchase this much over the course of phase one of the market trial.

27 Since arc elasticities involve discrete changes in prices and quantities, it is well known that a given change can yield different arc elasticities depending on the assumed direction of the change, which determines the denominator. In this case, we choose to frame this price change as a 50% reduction and therefore interpret the resulting arc elasticity as the sensitivity of demand to cutting the price of SQ-LNS in half. The comparable arc elasticity associated with a 100% price increase from low to high price effectively reduces the computed arc elasticities in half. We are especially interested in how the arc elasticity for repeat purchases compares to all purchases. This comparison of initial to repeat purchases is not sensitive to the assumed direction of the price change.

28 As another correction for displaced sales, we can restrict the analysis to the first four weeks of the trial. This yields estimates that are comparable, but less precise because of the smaller sample.

29 In creating these bootstrapped samples, we assume each purchase transaction is independent. We do not cluster these bootstrap samples by household in order to avoid over-weighting initial purchases relative to repeat purchases.

30 To appreciate this assumption, consider the following. If a household with a voucher booklet has a target age child, then weeks without an SQ-LNS purchase are true zeros from the standpoint of this study. Trusting the screening protocol used to distribute voucher booklets, we assume that households with a booklet have a target age child. Over the course of the study, however, it is possible that a child “ages out” of the target age range. For the voucher-level analyses, we assume that all non-purchases are zero purchases and not due to aging out of the target age range. This is a conservative approach that potentially suppresses the level of demand.

31 Results throughout the paper that cluster by village are largely qualitatively unchanged when clustering by village-month, which allows for spatiotemporal correlations in the standard error and increases the number of clusters (Cameron and Miller, 2015). While other adjustments to small clusters are possible (e.g., wild cluster bootstrapped standard errors), these adjustments can be modest in random effects models (Esarey and Menger, 2017).

32 Results throughout the paper that cluster by village are largely qualitatively unchanged when clustering by village-month, which allows for spatiotemporal correlations in the standard error and increases the number of clusters (Cameron and Miller, 2015).

33 Such tests of discrete habit formation that leverage randomizations have recently been conducted in the case of exercise in the U.S. (Charness and Gneezy, 2009; Royer et al., 2015).

34 Typically, the presence of a lagged dependent variable in a panel model introduces potential bias and prompts the use of dynamic panel estimators, but this potential bias fades as the number of time periods increases (Judson and Owen 1999). To ensure sufficient time periods to reduce this bias, we estimate this specification using data from the 32 weeks covered by phases one and two (with additional controls for phase two) in addition to estimating the model using the first 17 weeks covered by phase one.

35 We include random effects rather than fixed effects for households in order to estimate coefficients on time-invariant household variables such as voucher source. Results are qualitatively robust to the inclusion of fixed effects instead.

36 We use 400 replications to construct these RI p-values. To reflect the MPCR design of this experiment, we conduct this randomization inference using strata defined by village pairs.

37 This pattern of results would suggest that relatively poor households in this context may be price unresponsive because even at very low prices SQ-LNS may be unaffordable. The estimates are only borderline significant when standard errors are clustered by village, but insignificant based on RI constructed p-values.

38 In these survival models we focus on persistent supplementation using three-week moving averages, but recognize that these sufficiency thresholds also have a duration dimension (i.e., number of months above a given sufficiency threshold). With this important abstraction in mind, we interpret these results as a “best case” scenario that concentrates on week-to-week supplementation patterns.

39 Meredith et al. (2013) conduct a similar test by randomly distributing coupons to either the husband or the wife in treated households.

40 We use the term ‘father’ for simplicity. While most of the male caretakers in our sample – indeed in the region – are fathers, there are some uncles, brothers and grandfathers acting as caretakers as well.

41 A more direct test of the role that fathers play in the persistence of a household’s demand for SQ-LNS would be to focus on the sub-sample of auction participants who attended as a mother-father couple and test whether the father’s WTP in the auction has a bigger influence on subsequent demand in the market trial than the mother’s WTP. While this yields a sub-sample that is too small to provide much statistical power, we do find that the father’s WTP is a stronger predictor of subsequent SQ-LNS demand than the mother’s WTP.

42 We also replace village pair fixed effects with village fixed effects since the randomization in phase three is at the individual level.

43 We also replace village pair fixed effects with village fixed effects since the randomization in phase three is at the individual level. We replace week fixed effects with a quadratic time trend and post-promotion period dummies to indicate four week periods after the promotion. Since the loyalty card is intended to reward buyers for four weeks of purchases for a total of four rewards, we also interact these four-week period dummies with the loyalty card to test whether its effect fades over these time periods.

44 At the time phase three was implemented, the four rewards had a market value of 42% (soap and tea), 50% (sugar) and 66% (bowl) of the 600 CFA required to purchase four weeks of SQ-LNS at the price of 150CFA/strip.

45 In other settings, targeting of health products is much more important. For example, the delivery of a chlorine solution to treat drinking water benefits measurably from mechanisms that screen potential users so that those most likely to use the solution and least able to pay for it self-select into delivery (Dupas et al., 2016).
