# Supplementary material for: Household demand persistence for child micronutrient supplementation
Source: J Health Econ. 2018 Nov;62:147–64. doi: 10.1016/j.jhealeco.2018.09.010 (PMC6277815; doi:10.1016/j.jhealeco.2018.09.010)
Supplement: Supplementary file 1 [file mmc1.pdf]

Household Demand Persistence for Child Micronutrient Supplementation

For Online Publication: Supplementary Figures and Tables

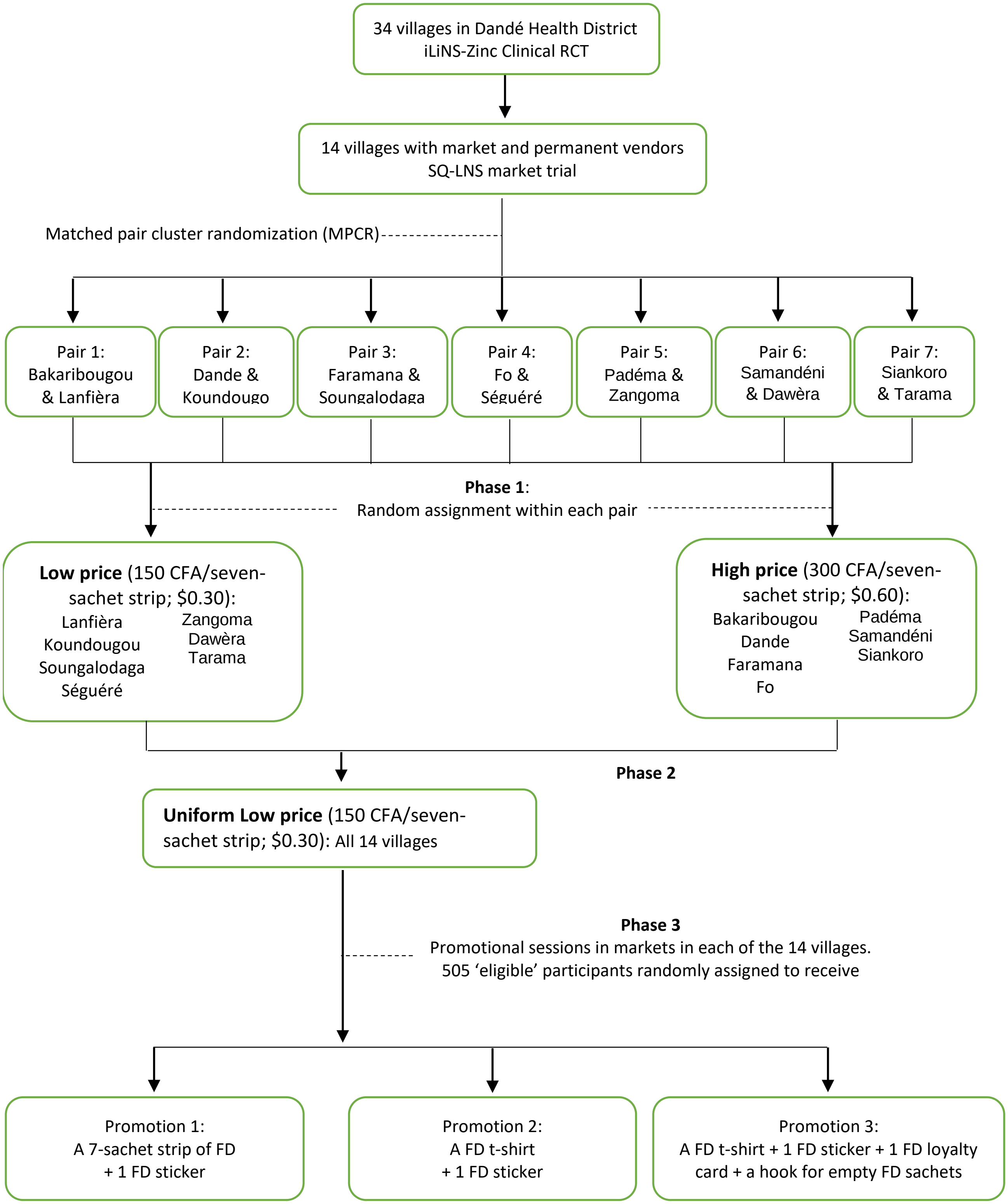

Figure S1 CONSORT diagram of research and randomization design of iLiNS nutritional trial and subsequent market trail.

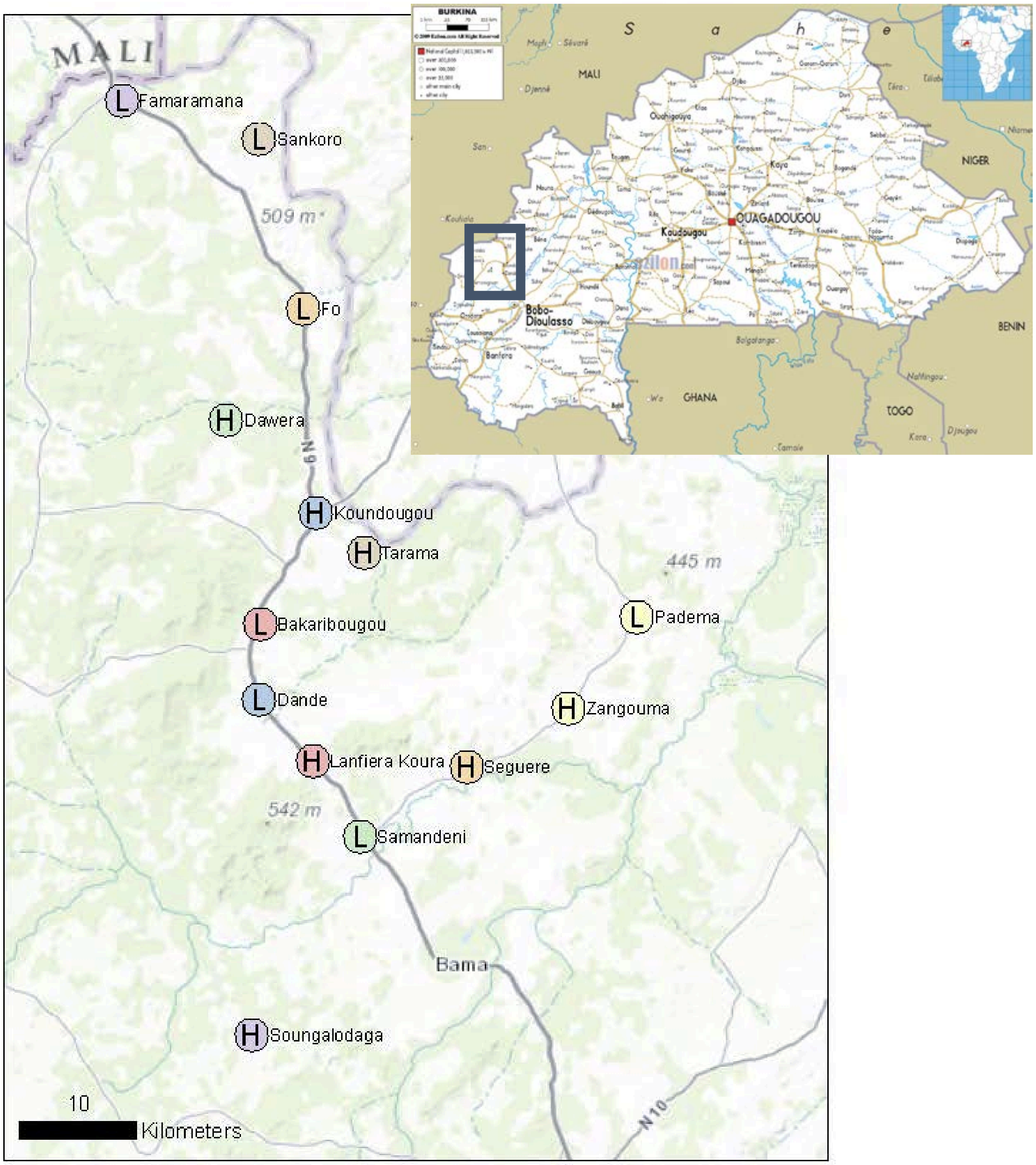

Figure S1 Map of villages in northwest Burkina Faso (north of Bobo-Dioulasso) included in SQ-LNS auction and market trial. Color of village dots indicates village pairs matched on relevant demand and market observables. Letters denote random high (H) and low (L) price assignment within pairs used during phase one of the market trial. At the conclusion of phase one (after week 17), SQ-LNS was priced in all markets at the low price.

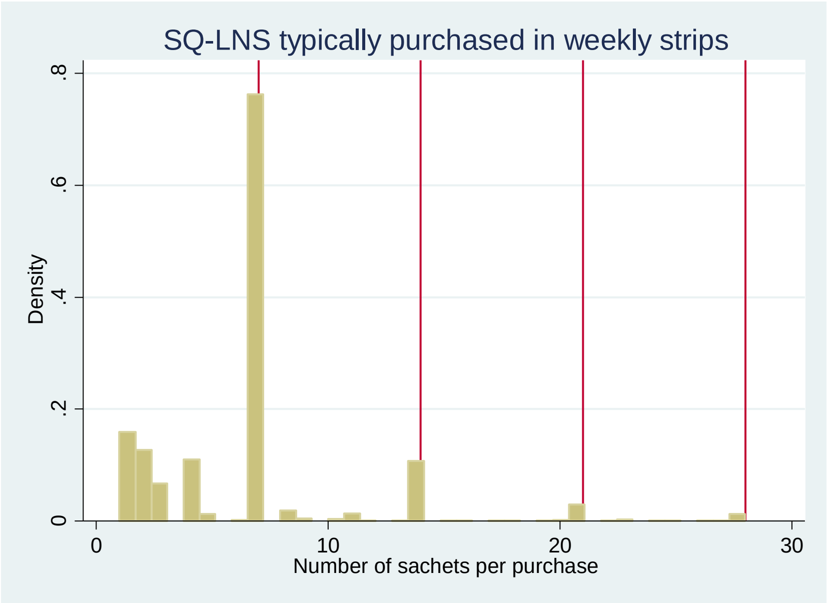

Figure S2 Number of sachets purchased per transaction with modes at multiples of weekly strips

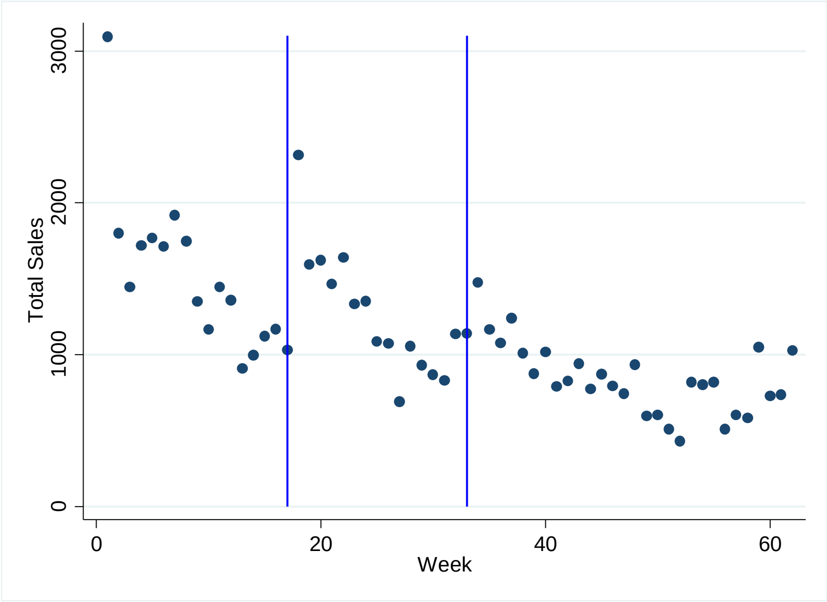

Figure S3 Total sachets of SQ-LNS sold in 14 market trial villages by week with vertical lines marking the transition from phase one (high-low prices by village) to phase two (uniform low price) to phase three (promotion and loyalty card).

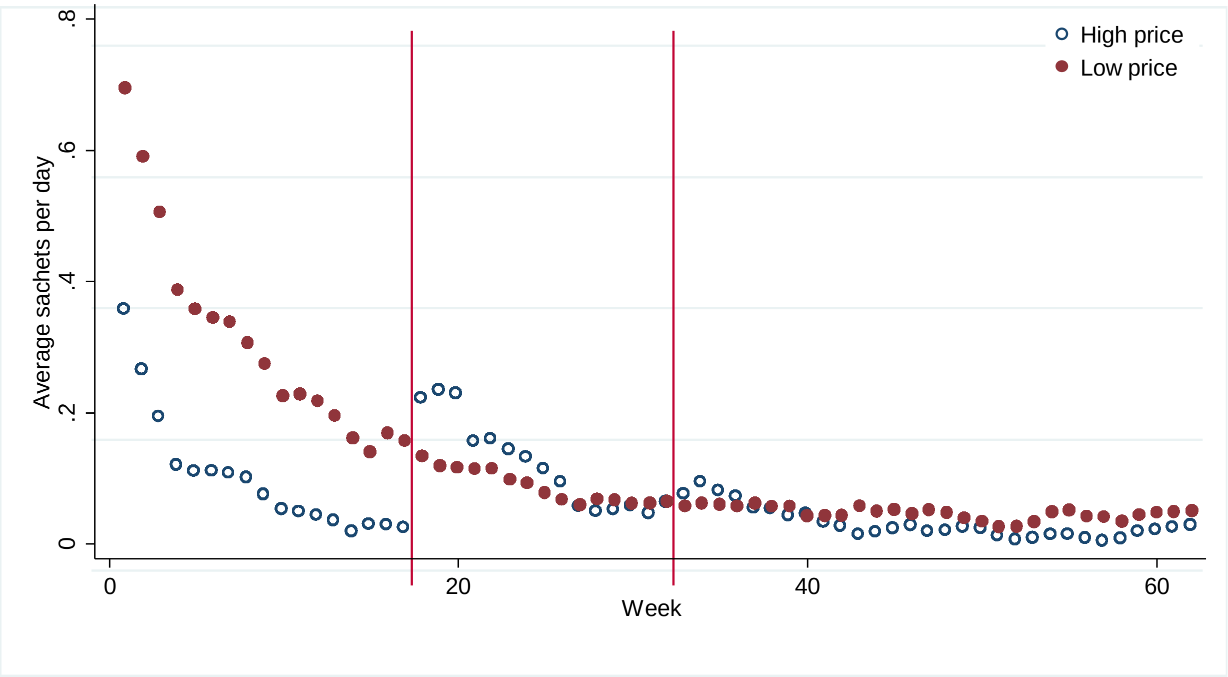

Figure S4 The average demand by week for initial high and low price villages. Weekly household-specific demand is measured as the three week moving average of sachets purchased per day. Vertical lines indicate the transition from phase one to two and two to three.

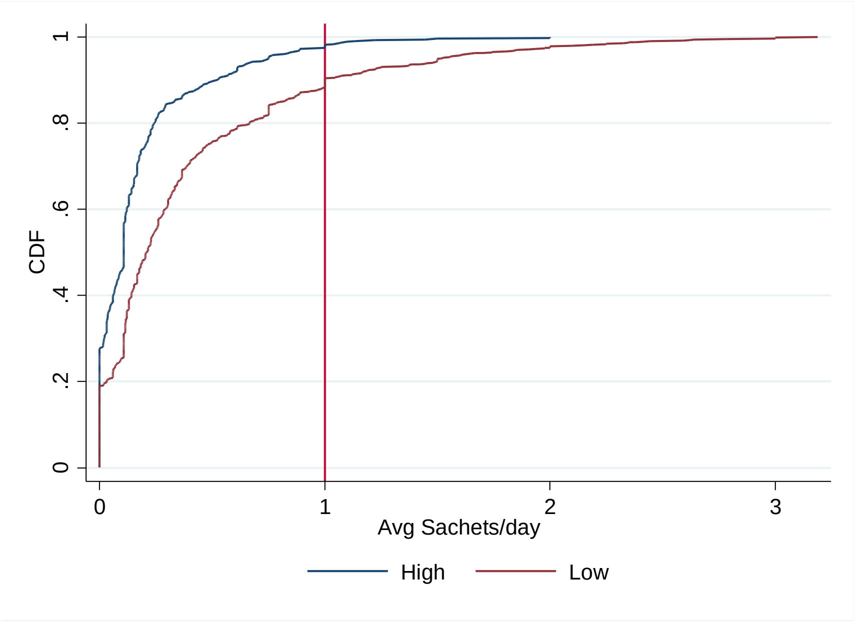

Figure S4 Cumulative distribution functions for average sachets purchased per day (three week moving average) by low price and high price treatment during phase one of market trial (weeks 1-17).

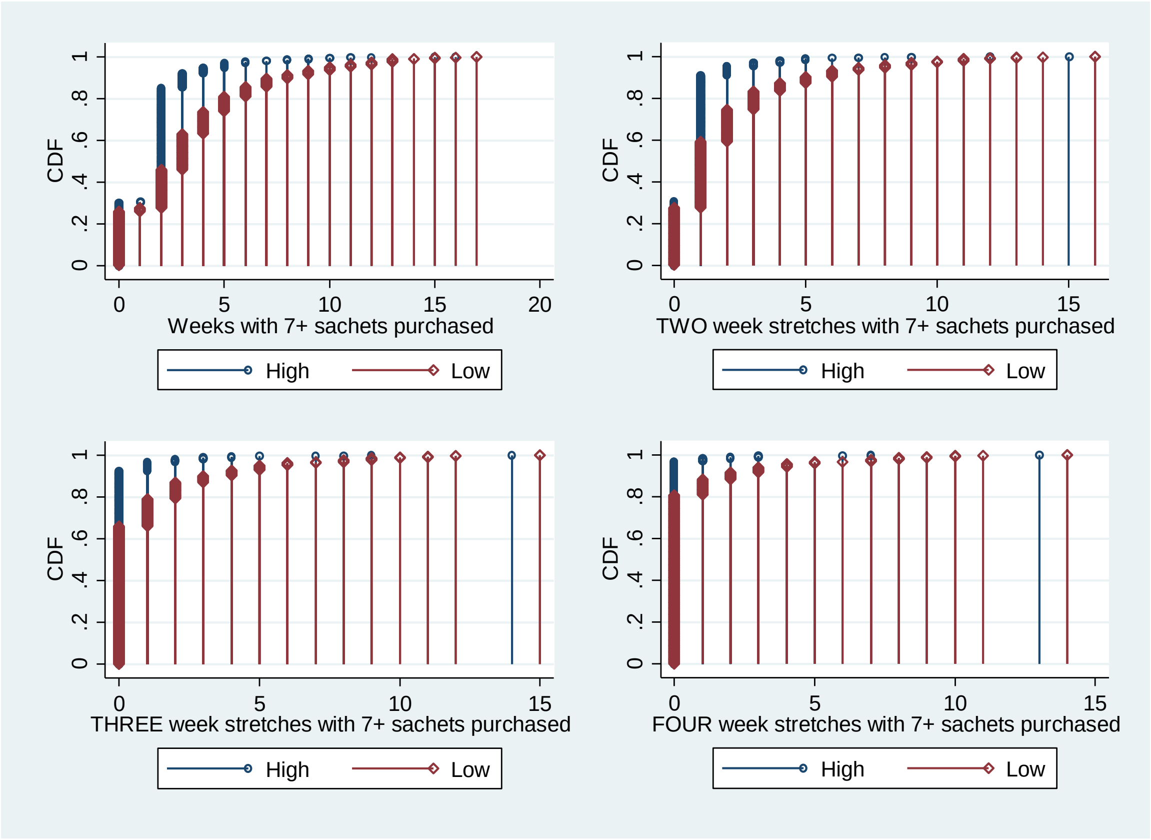

Figure S5 Cumulative distribution functions of consistent daily purchases by low price and high price treatment during phase one of market trial (weeks 1-17).

Table S1 Typology of differentiated LNS product classes that have emerged in response to the success of Plumpy'Nut® since the early 2000s.

	Intended purpose	Typical daily ration	Supply chain features
**Large-quantity LNS** (Ready-To-Use Therapeutic Foods (RUTF))	Treat severe acute malnutrition (SAM).	180-280g (1000-1500 kcal) to provide 100% of energy demands for 9-12 month old child outside of breast milk.	Private sector production with public sector procurement (UNICEF, MSF, WFP) and public distribution in collaboration with national health programs. Distribution through markets often illegal.
**Medium-quantity LNS** (Ready-To-Use Supplementary Foods (RUSF))	Treat moderate acute malnutrition and prevent SAM.	45-90g (250-500 kcal) to provide 50-100% of energy demands.	Private sector production with predominantly public sector procurement (UNICEF, MSF, WFP) and public distribution in collaboration with national health programs. *Expected: Some private sector distribution through markets.*
**Small-quantity LNS** (SQ-LNS)	Prevent undernutrition; promote normal growth and development.	20g (110 kcal) to provide <50% of energy demands.	Private sector production. *Expected: Sparse public procurement and distribution and active private sector distribution through markets.*

Source: (Arimond et al., 2013).

Table S1 Research design by phase and descriptive statistics disaggregated by voucher

			# Vouchers Distributed	# Active Vouchers	% Active Vouchers	Average Sachets per day	Source (% of voucher booklets distributed)									
							Auction (15%)		Auction + iLiNS Participant (15%)		Friend of iLiNS Participant (31%)		Vendor (28%)		Promotion (11%)	
							(1)	(2)	(1)	(2)	(1)	(2)	(1)	(2)	(1)	(2)
Phase 1	High price (H)		558	403	72%	0.13	57	32%	81	70%	190	100%	75	100%	-	-
	Low price (L)		913	740	81%	0.31	79	37%	122	75%	360	100%	179	100%	-	-
	Total		1,471	1,143	78%	0.25	136	35%	203	73%	550	100%	254	100%	-	-
Phase 2	H-L		859	467	54%	0.17	41	23%	76	50%	163	56%	186	78%	1	100%
	L-L		1,099	396	36%	0.11	32	15%	61	32%	148	35%	155	57%	-	-
	Total		1,958	863	44%	0.14	73	19%	137	40%	311	44%	341	67%	1	100%
Phase 3	H-L-L	(a)	78	47	60%	0.13	2	100%	8	53%	4	80%	5	83%	28	56%
		(b)	140	41	29%	0.03	4	40%	7	25%	5	42%	6	75%	19	23%
		(c)	982	374	38%	0.07	24	14%	38	28%	125	39%	185	53%	2	100
		Total	1,200	462	39%	0.07	30	17%	53	29%	134	39%	196	54%	49	37%
	L-L-L	(a)	79	41	52%	0.13	6	55%	9	60%	1	50%	2	50%	23	49%
		(b)	184	51	28%	0.04	4	27%	9	29%	8	57%	3	27%	27	24%
		(c)	1,183	355	30%	0.07	22	12%	39	22%	111	24%	182	50%	1	100%
		Total	1,446	447	31%	0.07	32	15%	57	26%	120	25%	187	49%	51	32%
	Total	(a)	157	88	56%	0.13	8	62%	17	57%	5	71%	7	70%	51	53%
		(b)	324	92	28%	0.03	8	32%	16	27%	13	50%	9	47%	46	24%
		(c)	2,165	729	34%	0.07	46	13%	77	25%	236	30%	367	51%	3	100%
		Total	2,646	909	34%	0.07	62	16%	110	27%	254	31%	383	51%	100	34%
Total	H		1,200	965	80.4%	0.10	78	44%	137	76%	336	99%	365	100%	49	37%
	L		1,446	1,158	80.1%	0.11	87	41%	172	78%	466	99%	382	100%	51	32%
	Total		2,646	2,123	80.2%	0.10	165	42%	309	77%	802	99%	747	100%	100	34%

Notes: (1) # active vouchers; (2) % of distributed vouchers in cell that are active. For phase 3, (a) with loyalty card, (b) without loyalty card, and (c) did not attend promotional meeting.

Table S3 Overall community compliance and coverage rate by village and by initial price treatment

Initial Price	Pair	Village	Population	Target-age children	Overall community compliance rate	Voucher coverage rate	Active voucher coverage rate
High	1	Bakaribougou	3,631	198	5.1%	77.8%	66.7%
	2	Dande	13,941	760	4.1%	47.5%	38.0%
	3	Faramana	8,463	461	3.9%	69.4%	55.5%
	4	Fo	3,562	194	6.6%	69.1%	51.5%
	5	Padéma	3,749	204	7.4%	78.9%	64.7%
	6	Samandéni	3,362	183	3.9%	93.4%	72.1%
	7	Siankoro	3,401	185	3.1%	100.7%	81.3%
Low	1	Lanfièra Coura	3,190	174	6.0%	82.8%	72.4%
	2	Koundougou	8,324	454	3.9%	75.6%	61.5%
	3	Soungalodaga	2,694	147	5.1%	89.8%	68.0%
	4	Séguéré	2,636	144	1.4%	35.1%	18.9%
	5	Zangoma	4,202	229	3.4%	84.3%	67.2%
	6	Dawèra	3,989	217	4.8%	97.7%	83.4%
	7	Tarama	2,461	134	5.0%	82.8%	67.2%
**Total**			67,605	3,684	4.5%	76.3%	61.6%

Notes: 'Pair' indicates village pairings used for MPCR.
Overall compliance rate is computed as (average sachets purchased per village per day / target-age children).
Voucher coverage rate is computed as (total number of voucher booklets distributed per village/ target-age children).
Active voucher coverage rate is computed as (total number of vouchers used per village / target-age children).

Table S4 The effect of observable household characteristics on demand for SQ-LNS based on endline sub-sample.

	Average sachets/day	# Weeks with sachets/day>0.3	Overall average >0.3 sachets/day	Total purchases <7 sachets
Household size	-0.00043	0.012	0.0012	0.0018
	(0.002)	(0.083)	(0.003)	(0.0061)
Asset index	0.028**	0.42	0.054***	-0.00084
	(0.011)	(0.410)	(0.016)	(0.024)
Hunger score	0.0076	-0.14	0.014	-0.13***
	(0.019)	(0.960)	(0.036)	(0.026)
Most of food is home produced	0.023	0.9	0.032	-0.021
	(0.028)	(1.510)	(0.040)	(0.060)
≤Half of food is home produced	0.057*	2.7	0.11**	0.015
	(0.028)	(2.090)	(0.050)	(0.083)
Low price village	0.017	-0.61	0.086*	0.069
	(0.029)	(1.460)	(0.044)	(0.055)
Father involvement index	0.063	4.01*	0.075	-0.13
	(0.048)	(2.200)	(0.081)	(0.092)
Constant	0.082***	6.97***	-0.012	0.34***
	(0.024)	(1.680)	(0.032)	(0.11)
Observations	335	335	335	335
R-squared	0.032	0.024	0.056	0.033

Robust standard errors clustered by vendor in parentheses.

*** p<0.01, ** p<0.05, * p<0.1

Appendices

Appendix A: The iLiNS-Zinc Clinical Nutritional Trial in Burkina Faso

Randomization for this clinical trial was done at the community and concession (i.e., family compound) levels. First, communities were stratified by selected indicators (population size; proximity to the all-weather road and to Bobo-Dioulasso; and health district affiliation) and then randomly assigned within strata into treatment communities (25) and control communities (9).[1] Children who met the inclusion criteria in the treatment communities were then randomly allocated to one of four intervention arms for 9 months (from 9 to 18 months of age): 1) SQ-LNS without zinc and placebo tablet (LNS-Zn0); 2) SQ-LNS with 5 mg zinc and placebo tablet (LNS-Zn5); 3) SQ-LNS with 10 mg zinc and placebo tablet (LNS-Zn10); or 4) SQ-LNS without zinc and 5 mg zinc tablet (LNS-TabZn5). Children in the control communities did not receive SQ-LNS or tablets from 9 to 18 months of age, but received SQ-LNS from 18 to 27 months after the data collection was finished. Enrollment of the rolling sample continued for approximately 11 months.

A weekly ration of SQ-LNS was delivered initially to participating children in the intervention groups in plastic pots containing 140g (sufficient for one week). The child's caregiver was provided with a measuring spoon and advised to feed the day's allotment (20g = 2 spoons) in two separate servings at mealtimes. After 13 months of project implementation, the packaging changed and children received seven sachets containing 20g each per week; caregivers were instructed to feed their children one sachet/day, mixed with food at mealtime. The SQ-LNS formulas for all treatment groups were identical, except for their zinc content. Zinc tablets were water-dispersible and contained 5mg zinc or an identical placebo. The caregivers were advised to provide the tablet once daily, dissolved in water or breast milk, but not with other foods. Caregivers were given brief child feeding advice at enrollment, which included the above described instructions for SQ-LNS and tablets and the recommendation to continue breastfeeding and to provide a large variety of foods. All children in the intervention communities were provided with continual monitoring for malaria and diarrhea, and treated as needed.[2]

[1] Treatment communities received SQ-LNS interventions at the outset of the study; control communities received SQ-LNS after the study had ended.

[2] Thus, the iLiNS-Zinc 'package' included home-delivered SQ-LNS, and continual monitoring/evaluation and treatment, as needed, for malaria and diarrhea.

Appendix B: Lab-in-Field Experimental Auctions for SQ-LNS

This appendix provides a summary of the experimental auctions that were designed to provide bounds on household demand for SQ-LNS. The detailed protocol is available upon request.

Auction participants were recruited via town crier the day before and the day of the auction. The crier announced the iLiNS team was in the village and wanted to speak with mothers and father of children age 6-24 months. Potential auction participants were screened for eligibility, and then eligible and willing participants received a participation fee (double the local daily wage rate) and were given some basic information about SQ-LNS and its potential benefits.

In short, participants were given an opportunity to purchase a week's supply of SQ-LNS for their children. Incentive-compatible WTP was elicited using a discrete version of the Becker-DeGroot-Marschak (BDM) mechanism in which participants were asked if they would be willing to pay specific, incrementally-increasing prices for a week's supply of SQ-LNS. Once a participant indicated s/he would not be willing to pay a specific price, his/her maximum WTP was recorded as the previous price in the series, and the participant purchased SQ-LNS if his/her maximum WTP was at least as high as a (subsequently revealed) 'market' price.

Because the efficacy of SQ-LNS likely depends on regular consumption throughout early childhood, we also asked a series of follow-up questions about WTP in the long-term. Specifically, we asked if a participant would pay his/her maximum WTP for SQ-LNS each week until his/her child was 24 months. The price was then increased/decreased in small increments, and the price at which the participant changed his/her answer was recorded as his/her long-term hypothetical WTP. Thus, this long-term WTP for SQ-LNS takes incentive-compatible demand as an anchor and prompts respondents to make adjustments away from this anchor in response to a hypothetical scenario. The goal of this mixed auction mechanism was to provide bounds on household WTP rather than precise estimates of demand.

Table B1 presents descriptive statistics of anthropometric, demographic, and economic data collected during these auctions.[3] Using these data, we explore the determinants of both incentive-compatible WTP for a week's supply of SQ-LNS and long-term hypothetical WTP. We model WTP for a week's supply using a Tobit maximum likelihood estimator, as the discrete BDM mechanism employed in the auctions means WTP is censored from above at the highest price in the price series. Long-term hypothetical WTP, which is not censored, is modeled using ordinary least squares. Although the series of auction sessions were designed to be as similar to one another as possible, small differences across sessions due to factors such as the composition of men and women, questions that arose during a session, or other session-specific factors could lead to correlation in bids among participants in a particular session. To account for this, standard errors are clustered at the auction session level (Cameron and Miller, 2015). Results are presented with and without auction session fixed effects in Table B2.

Across both specifications one and two in Table B2, incentive-compatible WTP for a week's supply of SQ-LNS is lower, all else equal, among households that previously participated in the iLiNS-Zinc clinical trial. All participants in the clinical

[3] Principal component analysis was used to combine household ownership of a set of assets[3] into an asset index (Vyas and Kumaranayake, 2006). Food security data were collected using an abbreviated version of the Household Food Insecurity Access Scale developed by USAID's Food and Nutrition Technical Assistance (FANTA) project (Coates et al., 2007). Each household received a food security score between 0-15 based on how frequently the household experienced each of five food insecurity conditions in the past four weeks, where higher scores indicate higher levels of food insecurity.

trial received SQ-LNS for free (either as part of the clinical trial or after the clinical trial as part of a control group), so these households had extended, first-hand experience with SQ-LNS prior to the auction. The negative association with WTP for SQ-LNS relative to participants who were not part of the clinical trial may be a reflection of these households' knowledge of the short-term private costs and benefits of SQ-LNS. Because SQ-LNS was provided for free to households who participated in the clinical trial, the negative relationship may also reflect a price anchoring effect whereby WTP is "anchored" to the previous price of zero.

Other statistically significant determinants of WTP for a week's supply are participant gender, weekly income, television ownership, and the asset index. WTP for SQ-LNS is higher, ceteris paribus, among male auction participants in the fixed effects specification, while the relationship between the household asset index and WTP is negative in this specification. Household ownership of a television is positively associated with WTP in both specifications. Participant income is negatively associated with WTP without fixed effects, but the magnitude of the effect is quite small.

Like WTP for a week's supply elicited from the auction, long-term hypothetical WTP for SQ-LNS is lower, all else constant, among households who participated in the clinical trial and higher among male participants. Household food insecurity is negatively associated with long-term WTP, where more food *in*secure households have a lower willingness to pay, all else equal. In specification three without fixed effects, the association between the age of the participant's youngest child and WTP is positive and significant.

Table B1 Descriptive statistics for households participating in the experimental auction.

	Variable	Definition	Mean/ Frequency	Std Dev/ Percent	Min, Max
Auction Participants	Male	=1 if participant is male	259	52.3%	
	Education	Years of education	1.9	1.9	0, 5
	Weekly Income	Self-reported income in past seven days (4th quarter 2011 USD)	22.89	72.1	0, 784.6
	Height	Height in meters	1.68	0.1	1.4, 1.9
	BMI	Body mass index (weight/height^2)	21.8	2.7	16.2, 34.3
Auction Households	Household Size	Number of household members	8.0	3.9	2, 27
	Child Age	Age of participant's youngest child in months	13.1	5.1	5, 24
	PC Weekly Food Expenditures	Per capita household expenditures on food in the past seven days (4th quarter 2011 USD)	1.22	2.6	0, 42
	TV	= 1 if household owns a television	91	18.4%	
	Asset Index	Proxy measure of household's socioeconomic status based on asset ownership	0.0	1.0	-4.5, 1.3
	Food Insecurity Score	Indicator of food insecurity in the household	1.8	2.4	0, 11
	Clinical Trial Household	= 1 if household participated in the iLiNS-Zinc clinical trial	103	20.8%	
WTP	Auction WTP	WTP for week's supply of LNS (4th quarter 2011 USD)	0.85	0.29	0.10, 1.18
	Auction hypothetical long-term WTP	Long-term WTP for week's supply of LNS (4th quarter 2011 USD)	0.75	0.51	0.10, 4.86

Table B2 Tobit regression results of WTP elicited in experimental SQ-LNS auction

	Variable	WTP for a Week's Supply (1) No Fixed Effects	WTP for a Week's Supply (2) Fixed Effects	Long-Term Hypothetical WTP (3) No Fixed Effects	Long-Term Hypothetical WTP (4) Fixed Effects
Participant Characteristics	Male (0/1)	0.0733	0.1042	0.1913***	0.2418***
		(0.0494) ‡	(0.0651)	(0.0611)	(0.0846)
	Education (yrs)	0.0088	0.0024	-0.0022	-0.0046
		(0.0110)	(0.0113)	(0.0148)	(0.0159)
	Weekly Income (2011 USD)	-0.0003*	-0.0002	-0.0001	-0.0000
		(0.0002)	(0.0002)	(0.0002)	(0.0002)
	Height (meters)	-0.2856	-0.1662	0.0196	0.1606
		(0.3306)	(0.3261)	(0.2962)	(0.2717)
	BMI	0.0024	0.0017	0.0097	0.0063
		(0.0077)	(0.0077)	(0.0085)	(0.0091)
Household Characteristics	Household Size	-0.0031	-0.0064	-0.0069	-0.0104
		(0.0049)	(0.0054)	(0.0050)	(0.0062)
	Child Age (mo)	0.0026	0.0006	0.0079**	0.0061
		(0.0033)	(0.0031)	(0.0033)	(0.0042)
	PC Weekly Food Expenditures	-0.0035	-0.0023	-0.0034	-0.0029
	(2011 USD)	(0.0061)	(0.0056)	(0.0069)	(0.0064)
	TV (0/1)	0.0963**	0.1073**	0.0692	0.1074
		(0.0444)	(0.0492)	(0.0719)	(0.0752)
	Asset Index	-0.0488*	-0.0663**	-0.0016	-0.0219
		(0.0258)	(0.0282)	(0.0327)	(0.0355)
	Food Insecurity Score	-0.0114	-0.0147	-0.0290**	-0.0307**
		(0.0100)	(0.0104)	(0.0118)	(0.0127)
	Clinical Trial Household (0/1)	-0.1023**	-0.0968**	-0.1711**	-0.1722**
		(0.0422)	(0.0443)	(0.0634)	(0.0695)
	Constant	1.2520**	1.0310*	0.3491	-0.8355*
		(0.5309)	(0.5531)	(0.4104)	(0.4208)
	Sigma	0.3915***	0.3753***		
		0.3915***	0.3753***		
	N	495	495	494	494
	Pseudo R^2/ R^2	0.028	0.085	0.084	0.155

Significance codes: *** (p < .01), ** (p < .05), * (p < .1)
Models specifications: (1) is tobit and does not include fixed effects; (2) is tobit and includes auction session fixed effects; (3) is OLS and does not include fixed effects; (4) is OLS and includes auction session fixed effects.
Note: Controls for market price in the practice rounds are included in all regressions (unreported).
‡Numbers in parentheses are robust standard errors, clustered at auction session level.

Appendix C: Detailed Description of SQ-LNS Market Trial

The market trial was carried out to estimate the demand for SQ-LNS (under the local name *Fanga Dengue* (FD)). A description of the market trial is presented in the following order: 1) vendors, 2) vouchers, 3) villages and prices, 4) participants, and 5) data collection. The vendor consent form, scripts, and other market trial data collection instruments are available upon request.

1. <u>**Vendors**</u>

Randomly selected vendors in 14 difference villages, of the 34 villages where the market trial was carried out, were invited to participate in the study as official vendor sites for FD. Out of all the invited, 29 official vendors sold FD during the 62 week trial.

The total number of sales by vendor during the entire market trial is presented in Figure C1.

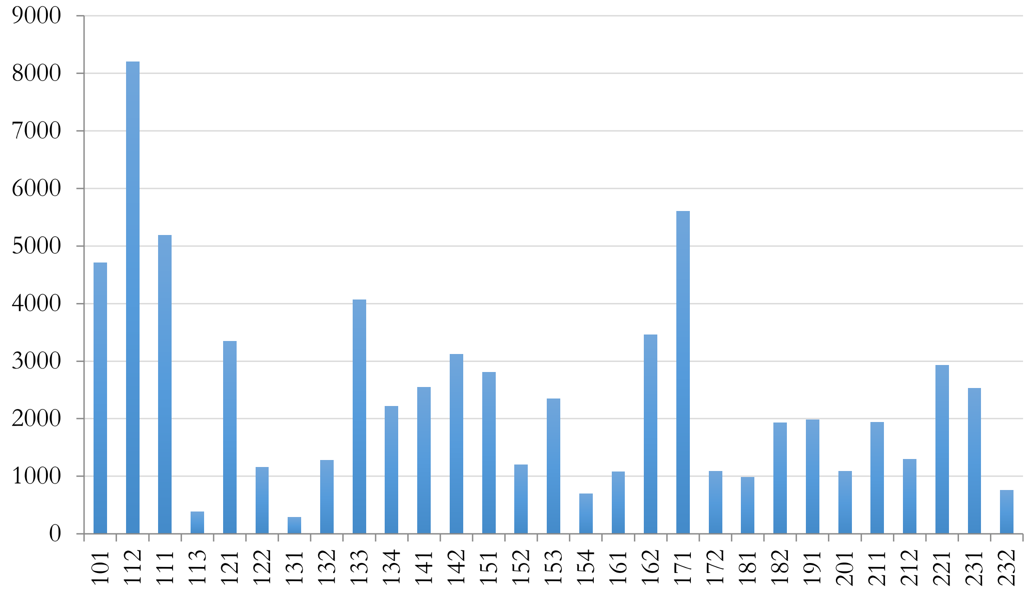

Figure C1: Distribution of sales by vendor during the entire market trial

Vendors were walked through a consent process in which they were assured that participation in the study was voluntary and they could withdraw their consent at any point in the study without penalty or negative consequences. If vendors were not able read or write, they were instructed to provide their consent using their fingerprint, at which point they received assistance from a witness, unaffiliated with the study, to explain the consent form and sign, verifying that the vendor understood the conditions and freely agreed to participate in the study.

Vendors who decided to participate had five main tasks:

a) Promote FD**:** to promote FD to people who come into the shop, each vendor received two posters to hang in the store and outside. The picture in the poster showed a mother and her two children representing the age range for the consumption of FD. Vendors emphasized the following main messages to potential buyers: 1) FD does not replace a diverse and nutritious meal; 2) a mother should breastfeed her child before giving him/her FD; 3) FD should be mixed with a small portion of the child's food; 4) FD promotes growth; 5) FD promotes good child

development; and 6) 1 bag, 1 child, 1 day. All this information was given in the form of “dominoes” that were distributed during the first purchase to a booklet holder.

Additional important messages that vendors knew and communicated to the potential buyers were: FD should not be given to a child under 6 months; it is not a medicine but a food supplement; reseal open bags and keep them in a clean place away from insects; FD should not be given to children allergic to milk (industrial or animal) or peanuts; and some children, when they first take FD, may have diarrhea. If the diarrhea persists for three days, they must stop consuming FD. After 3-6 days caregivers can start giving the child FD again and if the child has diarrhea again, caregivers must permanently stop giving FD to the child.

b) Sell FD at the price set by the FD team and enforce quantity limitations

The maximum quantity to be sold to an individual in a single transaction was a month’s supply (4 strips or 28 bags), and customers needed to provide a concrete justification for single transaction purchases greater than this (like traveling). Figure C2 shows that the mode of purchases was 7 sachets per week.

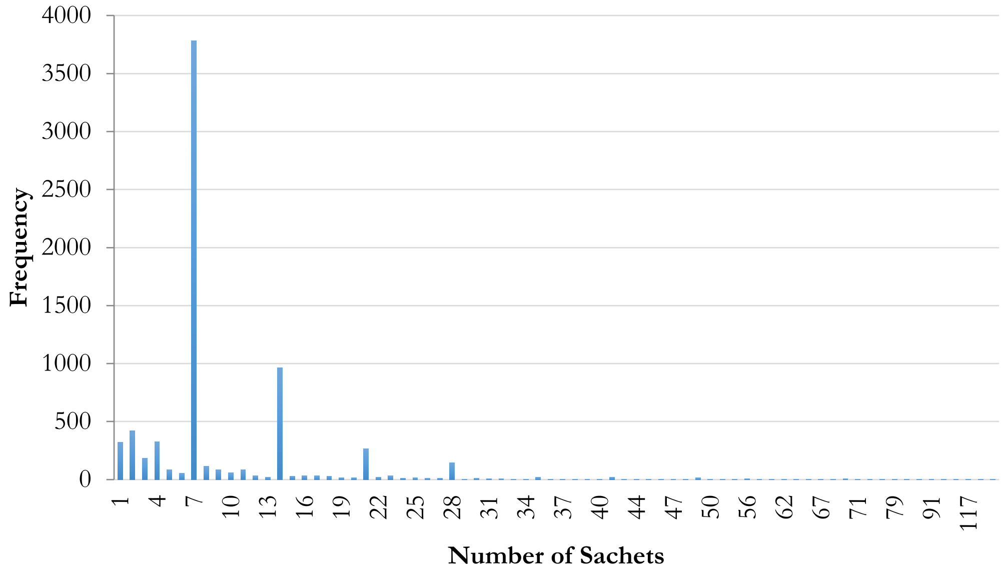

Figure C2. Number of sachets purchased per week conditional on positive purchases.

c) Distribute voucher booklets and collect a minimum of information on the people who receive them

Each vendor received a stack of voucher booklets to distribute to people that didn’t already have a booklet. When vendors issued a new booklet, the buyer’s name, village, district, phone number, and name of head-of-household or concession was recorded on the back of the first purchase coupon.

To track household purchases, each household had a unique number indicated on their voucher booklet, and vendors ensured each household received only one voucher booklet. Vendors were also responsible for informing the FD team when a buyer’s booklet was almost finished so that a new booklet with the same identifying number could be issued.

d) Collect coupons from buyers for every purchase of FD

Each time a customer purchased FD, vendors detached a page from the voucher booklet, recorded the amount of sachets of FD purchased, and kept the coupon in a lockbox that was provided to them.

The coupons that vendors kept allowed the FD team to count the amount of FD sold and to calculate the vendors' compensation. It was therefore very important that FD was not sold to people without a voucher booklet.

To facilitate the separation of monetary accounts of FD sales and other products in the store, a working capital of 300 CFA francs (in 25 or 50 francs) were given at the beginning of the sale to vendors. All sales had to be in cash (no credit).

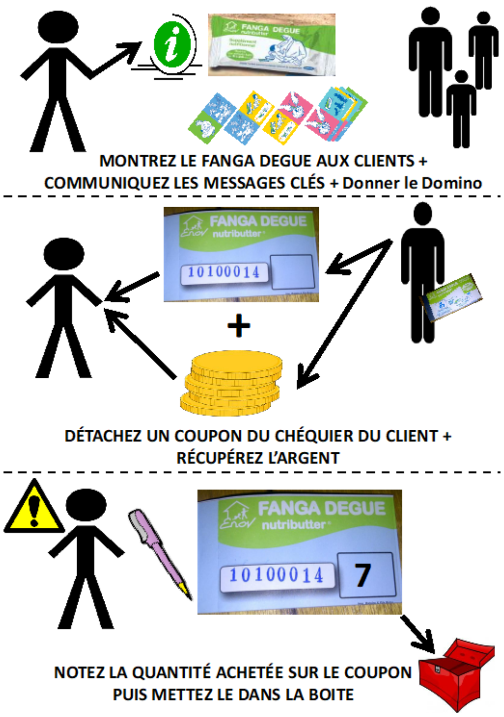

e) Collaborate with the FD team

The FD team visited vendors several times a week and were also available whenever vendors needed support. As compensation for their role in the market trial, vendors received 10 Frs. CFA (0.02 USD) for each sachet sold. This amount was the same for all the vendors regardless of the selling price in the village. Sellers received their compensation monthly, which was calculated from the accounts made twice per week by the FD team.

At each visit, the FD team collected and counted the revenue from the week's sales. After the first week, vendors were charged either 25 or 50 Frs. CFA for any missing sachets. The FD team checked that the revenue collected matched both the inventory count and the number of sachets sold as indicated on the coupons collected. At the end of the visit, the FD team made sure that the vendor was left with the appropriate change (300 Frs. CFA in small denomination).

2. <u>Voucher Booklets</u>

Each voucher booklet had a unique number that allowed the FD team to track household purchases.

Coupon numbering system: each coupon was marked with an eight-digit code, which identified the village (first two digits), source of the coupon (third digit), participant id (the following four numbers), and if the participant was a friend of an iLiNS participant (last digit). The last number had a number 1, 2 or 3, if the booklet was given to the 1st, 2nd, or 3rd friend of an iLiNS participant, respectively. If the booklet was acquired in any other way, this number was "4".The booklet's color was also linked to the source of the coupon: Auction participant (blue, #1), Auction & iLiNS participant (blue, #2), iLiNS participant (red, #3), iLiNS friend (red, #4), Volunteer (#6), and Promo (#6).

Figure C3 presents the distribution of the source of the voucher booklets that were used at least once. Figure C4 is an image a voucher booklet.

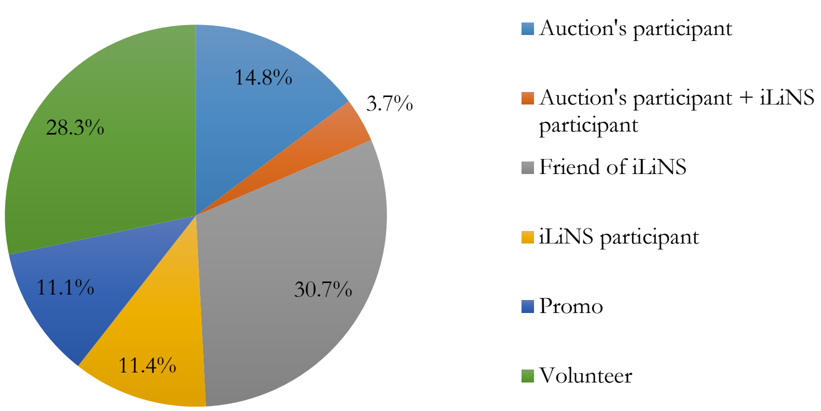

Figure C3. Share of voucher booklets used at least once by source

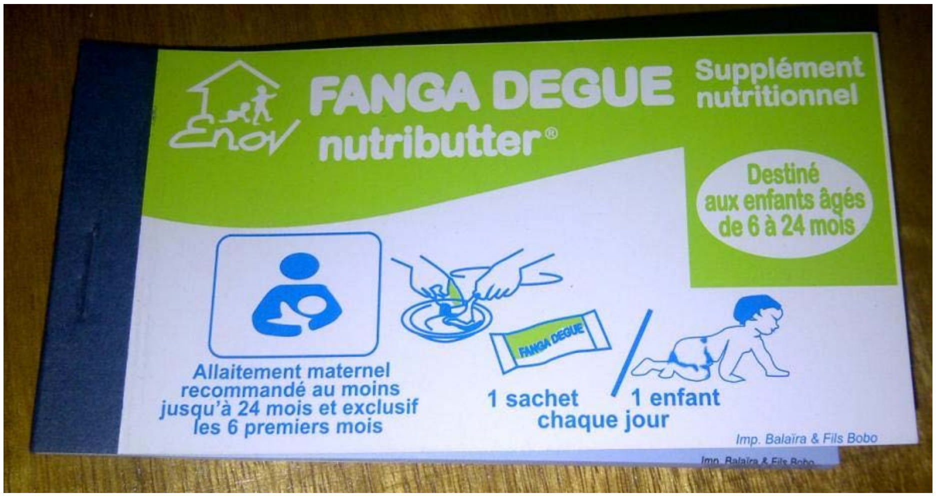

Figure C4. Sample voucher booklet

3. Villages and Prices

Out of the 34 villages that were part of the iLiNS-Zinc study, 14 villages were chosen to participate in the market trial based on the presence of permanent vendors and weekly markets in these villages: Bakaribougou, Dandé, Dawera, Faramana, Fô, Koundougou, Lanfiéra Koura, Padema, Samandeni, Siankoro, Séguéré, Soungalodaga, Tarama, and Zangoma.

Two different prices were charged in the 14 villages where the study was conducted in order to know if price differences have a significant impact on purchases. For the first 17 weeks of the trial, half of the villages (7) had a high price per set of 7 sachets of FD (300cfa or 50 Frs per sachet) and the other half had a low price per set of 7 sachets of FD (150cfa or 25 Frs per sachet). After the first 17 weeks, every village had the same price.

High- and low-price villages were randomly determined using a 'matched pair cluster randomization' (MPCR) approach. First, we matched villages by exploiting baseline census data, market characteristics data, and detailed socio-economic data that were collected in the broader iLiNS-Zinc project. Specifically, we selected six matching variables to capture features of demand and market size that are relevant to SQ-LNS: village population, distance from village to paved road, village mean asset index, village mean food insecurity score, mean number of shops per village, and the share of households that had heard of Plumy'Nut® prior to the beginning of the project in 2009. These six matching variables were used to construct a factor analytic matching index. Finally, we paired villages with their nearest neighbor in terms of this index.[4] With the village pairs formed, a simple coin toss determined which of these villages (vendors) would sell SQ-LNS at the low price (150 CFA/seven-sachet strip; $0.30) and which would sell at the high price (300 CFA/seven-sachet strip; $0.60). The villages with the low price were: Bakaribougou, Dandé, Faramana, Fô, Padema, Samandeni, and Siankoro.

4. Participants

Any person with a voucher booklet could go to an authorized vendor and buy FD at a determined price for 62 weeks. There were five ways to access a booklet: 1) Having participated in a FD auction during the month of June; 2) Having a child who participated in the iLiNS-Zinc study; 3) Being the parent/friend of the family who participated in the iLiNS-Zinc study, (3 checkbooks were given to each FD participant to distribute to their families/friends); 4) Via a promotional session; or 5) Directly from a vendor.

5. Data collection

Data collection began with the agents upon visiting the vendor. The agent collected and recorded information from the coupons, including the ID number, total number of sachets purchased, strips purchased, single sachets purchased, prices for strips and individual sachets, and the total price for the purchase. The agent totaled each of these columns on site then counted the inventory to address any discrepancies between the inventory and reporting.

Coupons were collected weekly or twice a week when the FD team visited the vendor as a part of the accounting process. The coupons, money, and forms were brought back to the office in Bama for data entry, review, and storage.

[4] To get unique pairs, we used an iterative process in consultation with our field manager. The final pairings are based on three first-order matches (nearest neighbors), one second-order match, one third-order match, and two fourth-order matches.

They also collected information from voucher booklets distributed by venders and renewals of booklets. For this, a notebook was available for the vendor to record information (names, phone numbers) which was later given back to the FD team as supporting evidence for the point of sales of FD and the calculation of the vendors' compensation.

Appendix D: Phase Three Promotional Sessions

In phase three of the market trial, which was launched in weeks 32-34, an individual-level non-price randomization was introduced that involved promotional sessions on market day in each of the villages. Data collection instruments and scripts used in association with the promotional sessions are available upon request.

The goal of the promotional session was to boost demand for FD and to test the impact of a non-price promotion (via a loyalty card program) on demand for FD. The promotional sessions were conducted during market days in each of the 14 villages with authorized vendors. For medium and large villages, several sessions (3-4) were conducted. For small villages, 2 sessions were given, or one "big" session with a small introductory session. Participants were invited by the town crier the night before the session with the following message: "The FD team invites the fathers AND mothers that have children who are between 6 and 24 months old to participate in a discussion session during the market day at ___ h." Groups of between 15 and 60 per village participated in the promotional sessions. The promotion team aimed to get roughly as many men as women to attend the promotions. Therefore, the crier was instructed to deliver the message and invitation to both fathers and mothers.

The sessions were organized as a discussion/information session with the majority of the participants sitting down in an arranged way to facilitate the delivery of the information. To accomplish this, each participant was greeted and seated, answered the enrollment questionnaire, and received a piece of paper with a number. Once every participant was accommodated, the presenter introduced FD in the form of questions-and-answers. For the first part, FD was presented to the audience and after that, 6 to 10 major questions were answered. The sessions were interactive, which meant that the presenter made sure that participants were listening and understanding the main points of the presentation.

During the information sessions, another team reviewed the enrollment questionnaires to identify targeted people who were eligible to spin the wheel on the basis of caring for a target age child. Eligible participants, most of whom were a mother or father of a target age child, then spun a wheel and publicly received one of three gifts:

1. A 7-sachet strip of FD + 1 FD sticker + 1 voucher booklet if he/she didn't have one.
2. A FD t-shirt + 1 FD sticker + 1 voucher booklet if he/she didn't have one.
3. A FD t-shirt + 1 FD sticker + 1 voucher booklet if he/she didn't have one + 1 FD loyalty card + a hook for empty sachets of FD.

Participants who won a loyalty card were eligible to redeem 28 empty sachets of FD to earn their choice of a reward. The card could be a maximum of four times over four months. The four different gifts available were a small sachet of tea, laundry detergent, sugar, and milk. We took a photo of those who won the loyalty card (after obtaining their consent) to facilitate monitoring the redemption of empty sachets for rewards. A copy of the photo was provided to them at the end of the activity.

Participants who spun the wheel were given a FD voucher booklet if their household did not already possess one and were asked a series of socioeconomic questions.

Appendix E: Endline Sampling Procedures

1. **Sampling procedure:**

The 375 participants who were part of the end-line survey were selected with the following sampling procedure:

- Buyers who made their first purchase after week 40 of the market trial were excluded from the sample.
- Remaining buyers were sorted from greatest to least by the ratio of total units bought and days as a client.
- The top 15% of buyers were identified and labeled with the letter A; the bottom 85% were labeled with the letter B.
- Out of all the buyers in this sub-population, 25% were chosen randomly. Group A was over-sampled to get more high-frequency buyers. The goal was for the "top 15% buyers" (A) to make up 25% of our sample and the "bottom 85% buyers" (B) to fill out the remaining 75% of the sample.
- The number of total participants in each village (N) was identified and also the number of participants in the top and bottom brackets. A pattern was design to randomly choose the participants (for example, take 1, skip 2, take 1, skip 3) and get the correct number of clients from each bracket in every village.
- All buyers who were friends of an iLiNS-Zinc participant were added to the sample (N=113). The original iLiNS household was not.

2. **Finding Clients/Recruiting**

Once the sampling was finalized, the FD team prepared a list of the selected buyers with all the information available from the voucher booklets (name, phone, location), iLiNS, auction records, promos, vendor supplied info, and the date of their last purchase. This was a tool to help agents for recruiting. The date of the last purchase was used to jog the memories of vendors: "this client purchased for the past 3 months" or "only last July," etc.

Many iLiNS households were located and asked about the voucher bookelts that they had distributed to their family and friends. They answered several questions, among them: "why these friends and not others?"; information of their friends like name, phone, location, relationship to iLiNS households, and their booklet numbers once they were located so FD team could link person/household to booklets.

Reasons for exclusion from sample: (N=46)

- Moved far away
- (For friends) iLiNS family moved away, no info on friends
- ILiNS family cannot remember who they gave booklet to
- The booklet got lost
- Child died
- Data entry error found regarding booklet number
- Vendor doesn't remember who client is
- Booklet was given back to vendor
- No information on booklet to begin search
- Lives in Ouaga
- Client had another booklet when found, replaced bad booklet number with one in hand, voucher data replaced in sample.
- Booklet registered to wrong person. If person found but not the booklet, that number was replaced in sample with one person is using/holding.

3. Data collection: (N=500)

- 6 agents and 12 assistants were recruited, hired, trained, and deployed for pilot testing between 7/24/2014-8/15/2014.
- Surveys were administered and data entered between 8/18/2014 – 9/12/2014.

Appendix F: Target Households & Assumptions About Non-Purchases

To leverage the richness of our voucher-level data, we must account carefully for non-purchases. To appreciate this issue and the assumptions in play, consider three types of household in our data. The first household type received a voucher booklet directly from our research team after being screened as having target-age children in either the auction or the promotional activities that mark the start of phase three. Such a household clearly qualifies as a 'target household' at the time of the screening. When the member of this household purchases SQ-LNS, the household's voucher number allows us to track this purchase. When the household chooses not to purchase SQ-LNS in a given week, we can infer this non-purchase is a zero purchase. After many weeks of non-purchases, however, we become less certain about how to treat non-purchases. All the information we conveyed to vendors and households emphasized that the target age window for children was 6-24 months. It is therefore possible that this household that is clearly in the target in the beginning ultimately 'ages out' of the target, in which case recording non-purchases as zeros may downwardly-bias estimated demand among the target population.

The second household type also received a voucher book from our research team after being screened as a target household, but never purchased SQ-LNS after receiving their voucher booklet. While the initial non-purchases may really indicate zero demand from a target household, our confidence in this inference ultimately fades. Moreover, our confidence that non-purchases are true zeros (purchases that should have taken place, but did not) fades more quickly for these household types than for the first household type because the lack of a track-record of purchases suggests the possibility that the household was screened into the target by mistake. In short, non-purchases by this second household type may indicate zero demand for SQ-LNS, but may also be due to 'aging out' (as before) or screening errors.

The third household type receives a voucher booklet directly from a vendor at the time of first purchase. Unlike the first and second household types, we have no initial screening to ensure this is a target household.[5] For this household type, then, we must rely entirely on the pattern of its observed purchases and non-purchases to infer whether it is truly a household in which a child of target age resides. For example, a one-off purchase without any repeat purchases may indicate that the household does not really have a target-age child, whereas sustained purchases over a few weeks may be strong evidence of a target-age child in the household.

As these three household types illustrate, we have two sources of information for inferring whether a given household has a target-age child or not: (i) our own screening procedures for the subset of households that received the voucher booklet directly from our research team and (ii) the purchase patterns of households.[6] This inference matters because it determines whether we treat a non-purchase as a zero purchase or as a missing value, which is a consideration for any voucher-level analysis. In contrast, the community compliance rates discussed above and presented in Figure 4 use village census data on the age distribution of children to determine the number of target-age children. These community compliance rates are therefore the most conservative estimates of 'coverage' by our market trial.

---

[5] We instructed vendors to share basic information about the proper usage and intended benefits of SQ-LNS to such customers, but have only indirect control over this information. In contrast, our research team had direct control over screening households in the auction and promotional activities (the first and second household types).

[6] As described above, we have some detailed data for a sub-set of households, including some household demographic data. Unfortunately, we do not have sufficient resolution on the age of target-age children to precisely determine when households 'age out'.

In order to proceed with voucher-level analyses, we formulate two inference approaches, one inclusive and the other more exclusive (i.e., restrictive). The inclusive approach conservatively assumes that all households that received their voucher booklets after being screened by the research team have at least one target-aged child throughout the market trial period. Among households that received their voucher booklets from a vendor, we assume that any household that makes more than one SQ-LNS purchase similarly has a target-aged child from the week of their first purchase until the end of the market trial. This inclusive approach results in a majority of non-purchases being registered as zero purchases for the entire market trial period.

The second, more exclusive approach imposes a series of criteria to infer whether a household likely has a child in the target age range. We use each household's pattern of SQ-LNS purchases to estimate the likelihood that the household has a child in the target age range and drop households when the likelihood of having a child in the target age range falls below 20%.[7] This approach is only viable for households with a purchase history. Households that received their voucher booklet in the auction or the promotional activity and that never made a purchase are dropped from the analysis after five weeks of non-purchase.

In the voucher-level analyses that follow, we use these two approaches as upper and lower bounds when assessing demand persistence. While the inclusive approach may downwardly-bias demand persistence, the exclusive approach may do the opposite. It is worth underscoring as well that these approaches obviously only apply to our voucher data; given that 'voucher coverage rates' are less than 100%, there are households with target-age children who never end up with a voucher booklet at all. As a comparison of these two approaches, Figure F1 shows a side-by-side progression of weekly demand persistence across the trial for low and high price villages by each approach. In the third phase of the trial, the exclusive approach to dealing with non-purchases leads to average demand that is roughly three times higher than the much more conservative inclusive approach.

[7] As outcome variable, this model uses a dummy variable that is one if the household's three-week moving average of SQ-LNS purchases is greater than zero in a given week and uses as four explanatory variables: (i) the cumulative total sachets purchased up to a given week, (ii) the total sachets yet to be purchased in future weeks, (iii) the number of weeks since last purchase, and (iv) voucher fixed effects. We estimate this as a linear probability model and adjust the predicted probabilities to be in the [0,1] range to facilitate the use of the criterion cutoff.

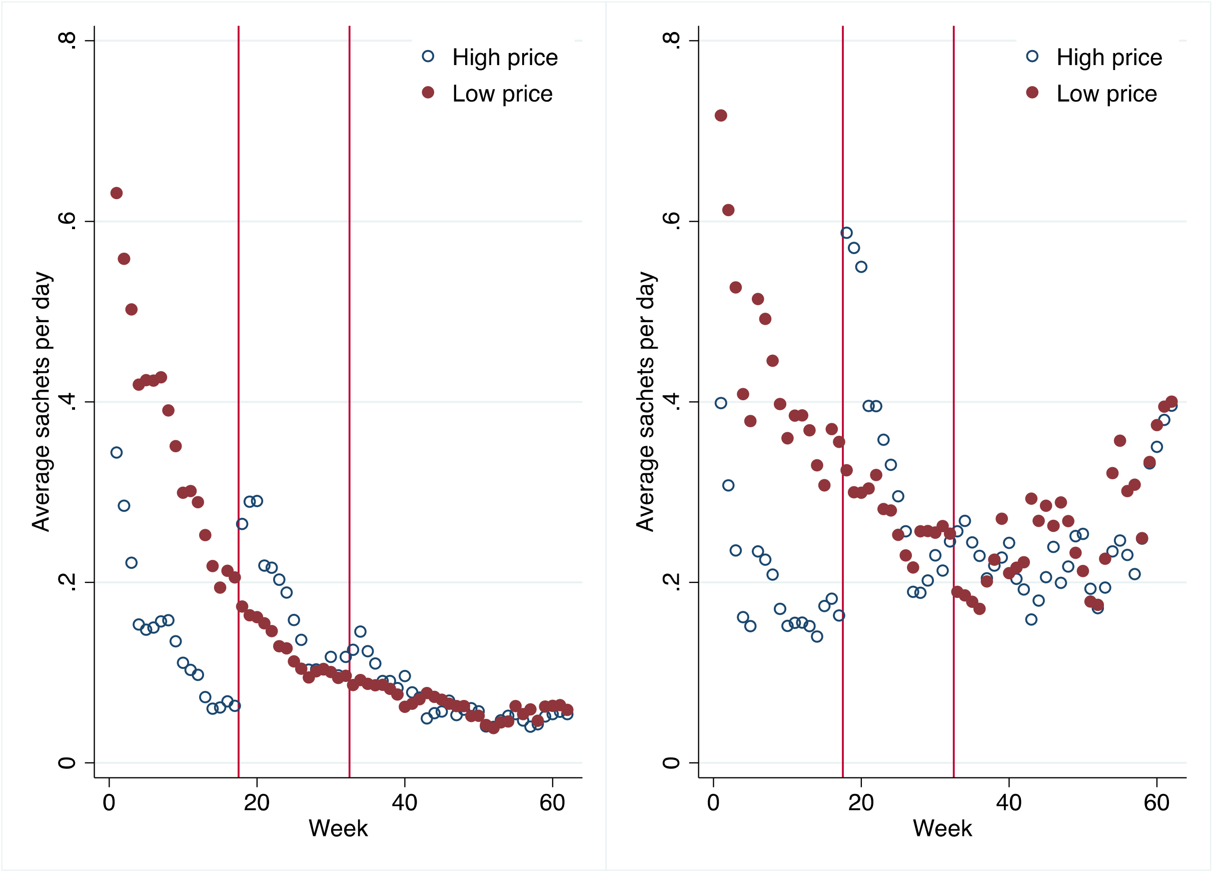

Figure F1 The average demand by week for initial high and low price villages. Weekly household-specific demand is measured as the three week moving average of sachets purchased per day. Vertical lines indicate the transition from phase one to two and two to three. The left panel uses the conservative 'inclusive' approach and the right panel uses the 'exclusive' approach to deal with weeks without a purchase.

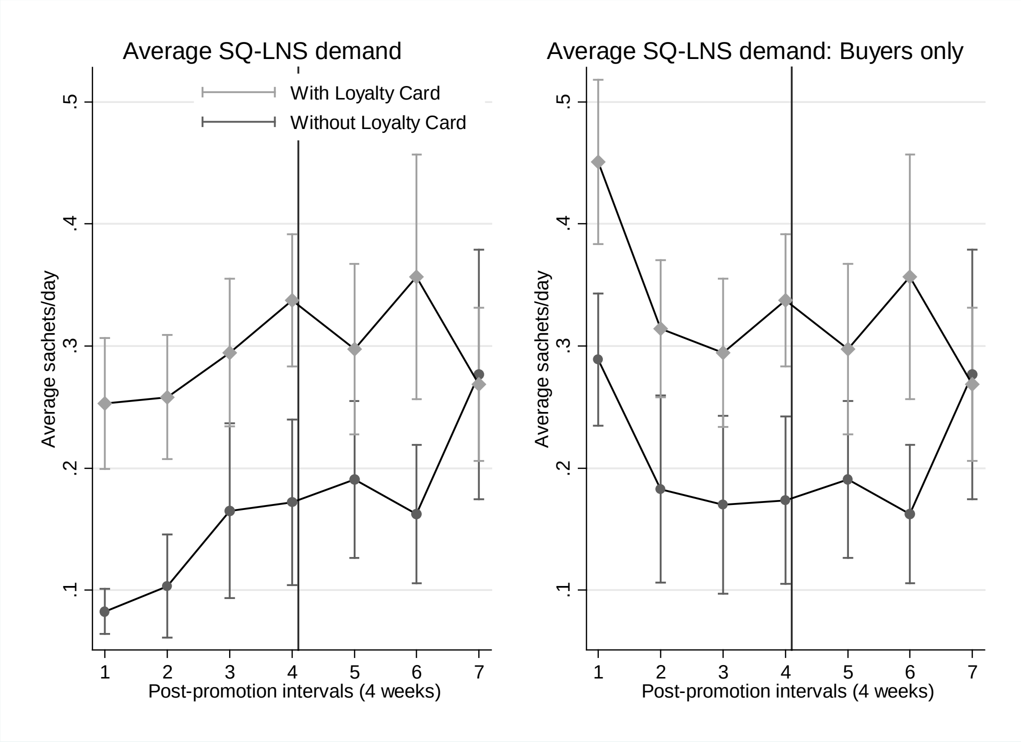

Figure F2 Conditional three week moving average of sachets purchased per day after promotional activities for households that won a loyalty card and those without a loyalty card, including all households that participated in the promotion (left) and only those that purchased at least one sachet of SQ-LNS after the promotion (right). Error bars depict 90% confidence intervals based on robust standard errors clustered by village. Vertical line indicates the earliest point that loyalty card holders could reach the maximum number of rewards. In contrast to the panels shown in the main paper, these use the 'exclusive' approach to deal with non-purchases.

Appendix G: Seasonality and Rainfall Effects on SQ-LNS Demand and Treatment Effects

SQ-LNS is designed to be consumed every day in order to meet shortfalls in micronutrient intake. For vulnerable households that experience seasonal fluctuations in food availability and/or household income, these shortfalls may be particularly acute at specific points during the agricultural cycle. Such volatility can directly hamper households' ability to invest in their children, including in the form of preventative health products such as SQ-LNS (Jensen 2000). Many households in our study area depend on rainfed agricultural as their primary source of income, and therefore seasonal variation in rainfall raises a particularly important dimension to household demand persistence for SQ-LNS in our sample. How much do agricultural production cycles and seasonal rainfall fluctuations shape demand for SQ-LNS in this context?

This question raises several potentially important considerations. Liquidity constraints, which fluctuate predictably according to the agricultural calendar and unpredictably according to production or market shocks, may hamper demand in lean seasons and may increase demand when good rainfall improves a household's realized harvest. Indeed, because rainfall realizations unfold gradually over the course of the production season, households are likely to adjust their demand based on intraseasonal changes in their expected end-of-season harvest. The production calendar also dictates investment of time, attention, and both purchased and non-purchased inputs. These seasonal investments may directly compete with SQ-LNS for scare household resources. To the extent that key production times are associated with the timing of rainfall, rainfall may drive much of this competition for resources. Finally, households may consider SQ-LNS to be particularly valuable during the lean season when diet diversity is low or even in anticipation of a harder-than-usual lean season (Arsenault et al. 2014).[8] To be clear, since the caloric and macro-nutrient (e.g., protein) content of each SQ-LNS sachet is very modest and expensive (per gram) relative to other foods, such a response would likely be due to expected micronutrient benefits rather than as a source of calories.[9]

In rural Burkina Faso, these seasonality effects have important health and nutrition consequences for children: During the lean season that accompanies later stages of agricultural production, children are both more vulnerable to disease (due to both less food availability and greater prevalence of diseases like malaria) and less likely to receive healthcare attention at rural health clinics because of the high opportunity cost of time and resources during that time (Sauerborn et al. 1996). While conducting explicit tests of these different

[8] Indeed, in some settings Doctors Without Borders has distributed SQ-LNS exclusively during lean seasons to prevent undernutrition – a form of temporal targeting.

[9] While we cannot rule out adults consuming SQ-LNS instead of target-age children, we encountered no evidence of this kind of leakage, which is not surprising given that far more cost-effective sources of calories and macronutrients are available locally. During the nutritional trial in our study area, however, sharing among children within a household occurred with some frequency.

potential pathways that link local rainfall to SQ-LNS demand persistence is beyond the scope of this analysis (and, indeed, our data), we nonetheless provide some initial evidence of how rainfall fluctuations, which are the basis for household expectations for end-of-season harvest in this rainfed production setting, shape demand persistence as supplementary analysis.

Phase one and much of phase three in our research design happen to coincide with the lean seasons of 2013 and 2014, respectively, which provides an opportunity to analyze how sensitive household demand for SQ-LNS is to rainfall during this particularly critical time of the year for child health and nutrition. While conducting explicit tests of these different potential pathways that link local rainfall to SQ-LNS demand persistence is beyond the scope of this analysis (and, indeed, our data), we can nonetheless provide some initial evidence of how rainfall fluctuations, which are the basis for household expectations for end-of-season harvest in this rainfed production setting, shape demand persistence. Because the sensitivity of end-of-season harvest to rainfall fluctuations varies widely depending on the timing within the production season, we estimate these effects separately for different sub-seasons.

To give some initial evidence in this regard, we collected rainfall data for our villages from the ARC2 dataset developed by the National Oceanic and Atmospheric Administration (NOAA) for programs of the U.S. Agency for International Development (USAID)/Famine Early Warning Systems Network (FEWS-NET).[10] We use these rainfall data to construct z-scores of weekly cumulative rainfall by village compared to long-run average rainfall for that week and village (see Figure G1).

Table G1 reports the results of a habit formation specification modified to include cumulative rainfall z-scores for phase one (late production season until harvest) and phase three (planting through late production season). The potential effect of rainfall on SQ-LNS demand via the mechanisms discussed above hinges on whether a given year is above or below normal rainfall. While interpreting these results, keep in mind that 2013 (2014) was significantly drier (wetter) than normal. We find a strong, positive effect of cumulative rainfall on SQ-LNS demand during the late production stage covered by phase one. Based on the estimated coefficient on the interaction of rainfall and the low price village dummy, demand is only sensitive to rainfall fluctuations in H-L-L villages – suggesting that rainfall shortages in 2013 significantly reduced SQ-LNS demand in these villages. In much wetter 2014, we see a much weaker rainfall effect on demand; indeed, the effect is negative and marginally significant in column four. We see habit formation as before, but rainfall does not

[10] The ARC2 dataset is derived from four sources: infrared satellite data to estimate cloud-top temperatures, ground-based rain gauge observations, and microwave SSM/I and AMSU-B satellite data. ARC2 offers the advantage of a very high spatial (0.1 deg x 0.1 deg) and temporal (daily) resolution, and is updated on a continuous basis. Validation of ARC2 with independent gauge data showed bias in certain regions and seasons, but an error rate that is comparable to other techniques (Novella and Thiaw 2013).

change this habit formation response. Results for endline survey households indicate that in 2013 wealth increased demand, especially in villages with better rainfall. In contrast, in 2014, household wealth had a direct negative effect on purchases for this subsample. While these results suggest a potential sensitivity of demand to rainfall in this semi-arid, rainfed setting, these are exploratory results given that the research design was not specifically intended to test these effects and the caveats mentioned above (e.g., the sequencing of these phases) apply here.

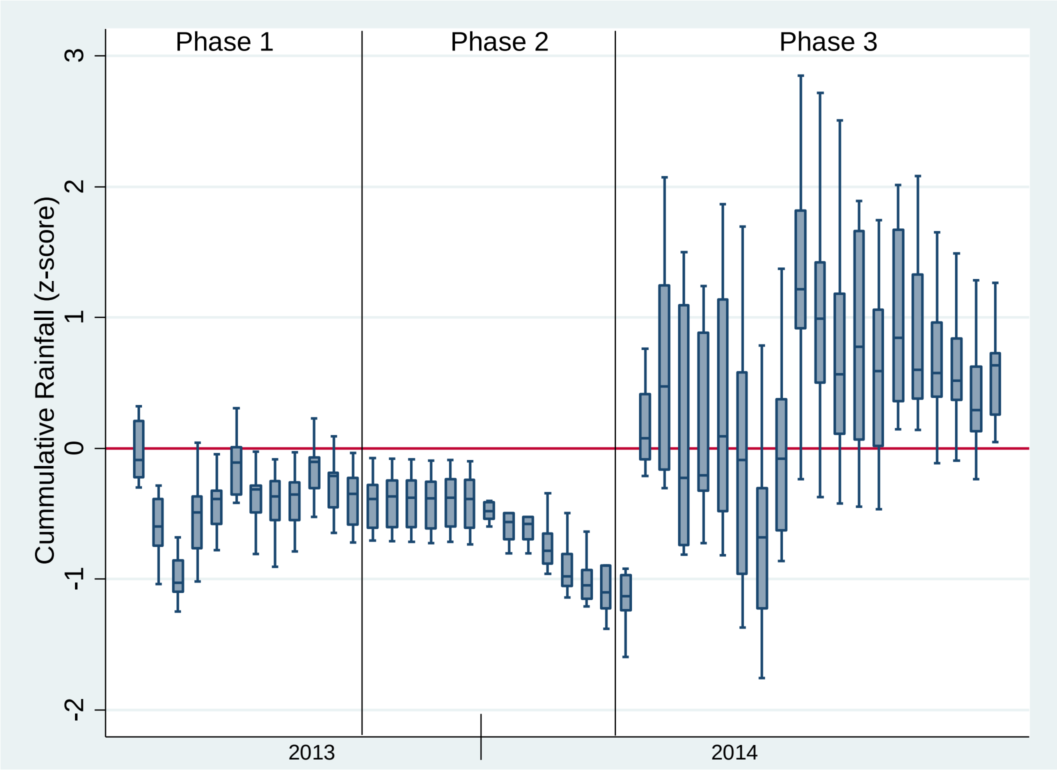

Figure G1 Box plots of cumulative rainfall relative to long-run average (z-scores) across 14 villages included in the market trial measured in 10-day intervals.

Table G1 Habit formation demand specification with household random effects and rainfall fluctuations (measured as z-scores relative to long-run cumulative rainfall by week) for phase one and phase three. Per Figure S6, 2013 was significantly drier than normal, and rainfall in 2014 was above normal.

	Phase 1: July-Nov 2013 [below normal rainfall]		Phase 3: Mar-Aug 2014 [above normal rainfall]	
	All	Endline Sample	All	Endline Sample
Low price village	0.168	-0.344		
	(0.170)	(0.292)		
Loyalty card			0.945***	1.074*
			(0.222)	(0.633)
Cumulative rainfall (z-score)	0.595***	0.326	-0.0644*	0.0787
	(0.203)	(0.562)	(0.0336)	(0.106)
…X Low price village	-0.688***	-1.640**		
	(0.255)	(0.735)		
…X Loyalty card			0.0328	-0.427*
			(0.0622)	(0.259)
Avg sachets (t-1)	0.0732*	0.257***	0.352***	0.218***
	(0.0388)	(0.0576)	(0.0455)	(0.0413)
…X Cum. rainfall	-0.0304		0.0598	
	(0.0575)		(0.0368)	
Asset index		0.285*		-0.485***
		(0.149)		(0.138)
…X Low price village		0.113		
		(0.260)		
…X Cum. rainfall		0.835**		
		(0.361)		
…X Low price village X Cum. Rainfall		-0.624		
		(0.824)		
…X Loyalty card				-0.180
				(0.447)
…X Cum. rainfall				-0.0925
				(0.0746)
…X Loyalty card X Cum. rainfall				0.00820
				(0.325)
Constant	0.538**	0.493	2.098	3.328
	(0.214)	(0.596)	(2.013)	(8.594)
Village FE	-	-	YES	YES
Village Pair FE	YES	YES	-	-
Observations	18,695	2,561	13,994	1,784
Number of vouchers	1,442	206	481	61

*** p<0.01, ** p<0.05, * p<0.1. Standard errors clustered by village-month in parentheses.

Unreported controls include week and week squared, voucher source, and - for phase 3 specifications - promotion dummies and post-promotion period dummies.

References

Arimond, M., Zeilani, M., Jungjohann, S., Brown, K. H., Ashorn, P., Allen, L. H., Dewey, K. G., 2013. Considerations in developing lipid-based nutrient supplements for prevention of undernutrition: Experience from the International Lipid-Based Nutrient Supplements (iLiNS) Project. Maternal & Child Nutrition.

Cameron, C. A., Miller, D. L., 2015. A practitioner's guide to cluster-robust inference. J. Human Res. 50(2): 317-372.

Coates, J., Swindale, A., Bilinsky, P., 2007. Household food insecurity access scale (HFIAS) for measurement of food access: Indicator guide, available.

Vyas, S., Kumaranayake, L., 2006. Constructing socio-economic status indices: How to use principal components analysis. Health Policy Plann. 21(6): 459-468.
